# Deciphering the regulatory and catalytic mechanisms of an unusual SAM-dependent enzyme

**DOI:** 10.1038/s41392-019-0052-y

**Published:** 2019-05-24

**Authors:** Qiu Sun, Yuehong Hu, Yijun Gu, Jiangkun Huang, Jun He, Lan Luo, Yi Yang, Shuo Yin, Chao Dou, Tianqi Wang, Xianghui Fu, Ling He, Shiqian Qi, Xiaofeng Zhu, Shengyong Yang, Xiawei Wei, Wei Cheng

**Affiliations:** 1 Division of Respiratory and Critical Care Medicine, Center of Infectious Diseases, National Clinical Research Center for Geriatrics and State Key Laboratory of Biotherapy, West China Hospital of Sichuan University and Collaborative Innovation Center of Biotherapy, Chengdu, 610041 China; 2 Shanghai Synchrotron Radiation Facility, Zhangjiang Lab, Zhangheng Road 239, Pudong District, Shanghai, 201203 China; 3 Department of Medicinal Chemistry, Key Laboratory of Drug-Targeting and Drug Delivery System of the Education Ministry, Sichuan Engineering Laboratory for Plant-Sourced Drug and Sichuan Research Center for Drug Precision Industrial Technology, West China School of Pharmacy, Sichuan University, Chengdu, 610041 China; 4 West China School of Public Health, Sichuan University, Chengdu, 610041 China

**Keywords:** Structural biology, Structural biology

## Abstract

*S*-adenosyl-1-methionine (SAM)-dependent enzymes regulate various disease-related behaviors in all organisms. Recently, the leporin biosynthesis enzyme LepI, a SAM-dependent enzyme, was reported to catalyze pericyclic reactions in leporin biosynthesis; however, the mechanisms underlying LepI activation and catalysis remain unclear. This study aimed to investigate the molecular mechanisms of LepI. Here, we reported crystal structures of LepI bound to SAM/5′-deoxy-5′-(methylthio) adenosine (MTA), *S*-adenosyl-homocysteine (SAH), and SAM/substrate states. Structural and biochemical analysis revealed that MTA or SAH inhibited the enzyme activities, whereas SAM activated the enzyme. The analysis of the substrate-bound structure of LepI demonstrated that this enzymatic retro-Claisen rearrangement was primarily driven by three critical polar residues His133, Arg197, Arg295 around the active site and assisted by SAM with unclear mechanism. The present studies indicate that the unique mechanisms underlying regulatory and catalysis of the unusual SAM-dependent enzyme LepI, not only strengthening current understanding of the fundamentally biochemical catalysis, but also providing novel insights into the design of SAM-dependent enzyme-specific small molecules.

## Introduction

Antibiotic resistance is a major concern worldwide, and new therapeutic drugs are urgently needed.^1–3^ Fungi are an invaluable resource of antibiotic candidates.^4–7^ Pyridone alkaloids (e.g., PF1140, leporins, and fusaricides) are present in numerous fungi, displaying diverse biological activities and applied for various pharmaceutical purposes.^8,9^ Owing to the complexity of pyridone alkaloids with a chiral center and pure forms, the efficient synthesis of these compounds has drawn increasing attention from chemists and biochemists.^10–14^ Pericyclic reactions are facile access to obtain various biomolecules including some pyridone alkaloids and have been considered as a key transformational process in the biosynthesis of many natural compounds.^15–18^ However, enzymes catalyzing pericyclic reactions rarely exist naturally.^19–21^ Recently, the novel multifunctional *S*-adenosyl-1-methionine (SAM)-dependent enzyme LepI, located in the *PKS-NRPS* gene cluster for leporin biosynthesis, was characterized in *Aspergillus flavus.*^22,23^ Ohashi et al. first reported this multifunctional enzyme in catalyzing pericyclic reactions for leporin biosynthesis.^22^ LepI is considered as a SAM-dependent methyltransferase that can catalyze stereoselective dehydration via three pericyclic transformational processes: intramolecular Diels-Alder and hetero-Diels-Alder reactions and a retro-Claisen rearrangement.^22,24^

The detailed mechanisms of SAM-dependent enzymatic actions are well understood,^25,26^ whereas the mechanism underlying LepI-catalyzed pericyclic reactions remains unclear.^22,24^

This study aimed to investigate the mechanism of LepI action. Herein, a series of structures, including those of SAM/5′-deoxy-5′-(methylthio) adenosine (MTA)-bound LepI, *S*-adenosyl-homocysteine (SAH)-bound LepI, and SAM/substrate-bound LepI, and various biochemical analyses helped elucidate the mechanisms underlying LepI activation and catalysis. The present study not only elucidates the novel mechanism of SAM-dependent enzymatic action but also provides potential insight regarding similar enzymes involved in various physiological processes. The findings of this study will facilitate the development of new enzymes for applications in chemistry and synthetic biology.

## Results

### Similar chemicals regulate LepI activity

Although pericyclic reactions are indispensable for the biosynthesis of many natural compounds,^20^ enzyme-catalyzed pericyclic reactions are rare in nature. LepI, an unusual SAM-dependent enzyme, can catalyze pericyclic transformations involved in the retro-Claisen rearrangement^22^ (Supplemental Fig. 1). However, the mechanisms underlying LepI activation and catalysis remain unclear, although LepI shares considerable sequence identity within the SAM enzyme superfamily (Supplemental Fig. 2).

**Fig. 1:**
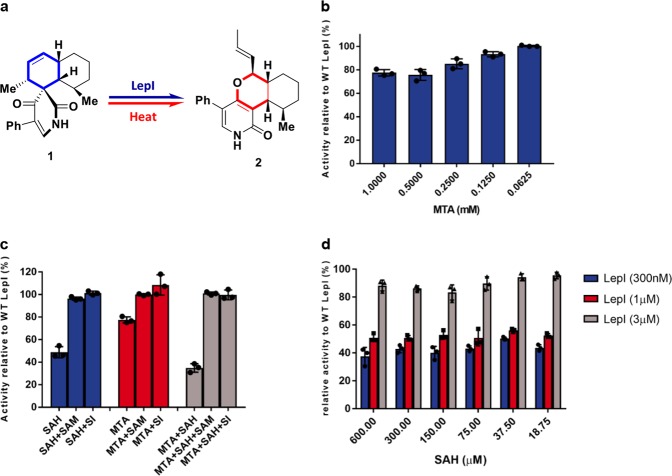
Similar chemicals regulate LepI activity. **a** Scheme for LepI-catalyzed retro-Claisen rearrangement. The structures show the relative stereochemistry. **b** Inhibition of MTA on LepI-catalyzed retro-Claisen rearrangement under different concentrations in the presence of 300 nm LepI. **c** Recovery of retro-Claisen rearrangement by 1 mm SAM or SI in the presence of 1 mm SAH or 1 mm MTA and 300 nm LepI. The experiments in **b**–**d** were performed three times, each with three biological replicates. Data are the mean ± S.D. **d** Inhibition of SAH on LepI-catalyzed retro-Claisen rearrangement under different concentrations in the presence of 0.3, 1.0, or 3.0 µm LepI

In this study, we performed an enzymatic assay using LepI to catalyze compound **1** to leporin C (Fig. 1a, Supplemental Fig. 1). Because some endogenously bound MTA and SAM copurified with LepI (Supplemental Fig. 3), we examined LepI enzymatic activity in the presence of MTA and simultaneously assessed LepI activity using the competitive inhibitor SAH.^27^ MTA moderately decelerated the retro-Claisen rearrangement of LepI when converting compound **1** to leporin C, decreasing its activity by 30% at 0.5 mm (Fig. 1b). Notably, MTA inhibited LepI activity in a concentration-dependent manner (Fig. 1b), whereas SAH decreased LepI activity by 50% in a dose-independent manner, slightly altered at enzyme concentrations of 0.3 µm and 1.0 µm, and even those approaching 3.0 µm (Fig. 1d). Remarkably, no inhibition was observed at a LepI concentration of ~3 µm. Intriguingly, the synergistic effect of MTA in the presence of SAH decreased LepI activity by ~ 70% (Fig. 1c). Furthermore, we investigated whether the positively charged SAM and the corresponding analog Sinefungin (SI) could rescue LepI activity when the enzyme was treated with MTA, SAH or both. Consistent with previous report,^22^ both SAM and SI completely rescued LepI activity for retro-Claisen rearrangement (Fig. 1c). This result prompted the following questions: why do MTA and SAH have synergistic effects? What are the different mechanisms underlying MTA and SAH inhibition? How can SAM/SI rescue LepI activity? We postulated that the mechanisms underlying activation or inhibition by these compounds could be distinct from those of other members of the SAM-dependent enzyme superfamily; this postulation was supported by structural and biochemical findings.

### Structures of the LepI complex

**Fig. 2:**
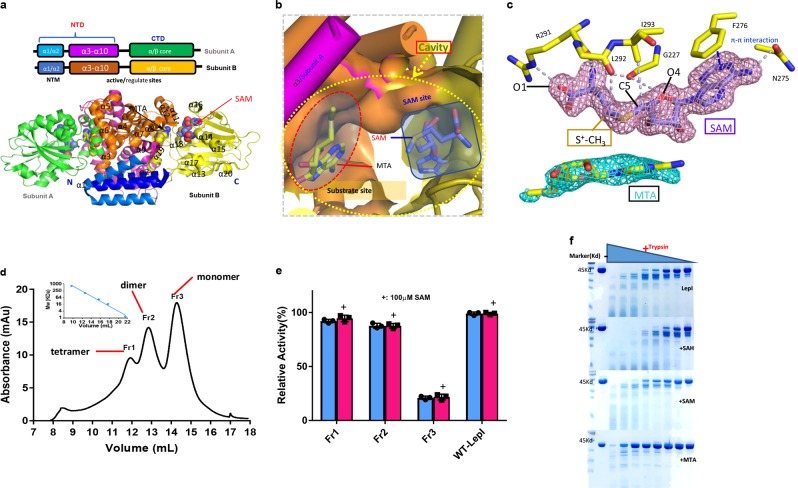
Structure of LepI in complex with MTA and SAM. **a** Architecture of the LepI dimers in complex with SAM and MTA. LepI adopts a SAM-dependent MT fold. Small molecules are indicated as spheres in the cavity of LepI structure. **b** The cavity comprises two sites: a SAM site and a substrate site. The close-up stereo view of the mimic substrate MTA and SAM-binding site indicates that the SAM (indicated by the red circle) site is independent of the substrate site (indicated by the green rectangle). **c** The 2*F*o–*F*c electron densities for MTA (colored cyan) and SAM (colored pink) at 1σ and 2σ, respectively. A close-up view of the detailed interaction between SAM and LepI is shown; gray dashed lines indicate the hydrogen bonds, and the blue dashed line indicates the π–π interaction. **d** Analytic gel-filtration of purified LepI-Δ15. Three peaks appear, representing the formation of LepI monomer, dimer, and tetramer according to the standard protein marker. A representative image from three replicate experiments is shown. **e** Enzymatic activity of LepI-Δ15 Fr1-Fr3 compared with wild-type (WT) LepI determined through retro-rearrangement assay with triplicate measurement. (Data represent the mean ± s.d.) The Fr3 monomer indicated in the gel-filtration assay is almost inactive, whereas both Fr1 and Fr2 still have full activity. **f** Limited proteolysis of LepI in the presence of SAM, SAH, or MTA at gradient concentrations of trypsin. The proteolytic fragments were detected by SDS-PAGE and Coomassie staining

To elucidate the mechanism of LepI action, we first determined the structure of SAM/MTA-bound LepI at a high resolution of 1.7 Å (Fig. 2a, b, Extended Data Table 1). The overall structure was found to be largely similar to that of SAM-dependent enzymes (Supplemental Fig. 4a–d),^28–31^ exhibiting a core α/β fold (Supplemental Fig. 4e) of alternating β strands (β1–β7) and α helices (α1–α19) (Fig. 2a). This structure exhibiting α helices and β sheets comprises an all-helix amino-terminal domain (NTD), a carboxy-terminal domain including a substrate-binding site, and a SAM-binding site (Fig. 2b, Supplemental Fig. 5), which is formed by a seven-stranded β sheet rounded with five helices. Two molecules of LepI observed in an asymmetric crystal unit form the homodimer, which is primarily mediated by the NTD (Supplemental Fig. 5a); specifically, the α1 and α2 segments of one subunit interact with those of another subunit to form interlocking fingers (Supplemental Fig. 5b), and α3 is involved in the formation of the hind surface of the active site (Supplemental Fig. 5c). To verify the importance of α1 and α2 in the dimers for enzymatic activity, we performed an enzymatic assay with α1 single deletion or α1–α2 double deletion of LepI. These deletions, particularly α1–α2 double deletion, completely obliterated the enzymatic activity (Fig. 2d, e, Supplemental Fig. 5d, e). Moreover, the α1 single deleted enzyme displayed stable variable conformations in a size-exclusion chromatographic assay. We then comprised enzymatic assays using each corresponding fraction (Fig. 2d, e). These results indicate that the α1 and α2 segments play an important role in LepI homo-oligomerization, which is essential for its activity.

As determined from the strong electron density, SAM binds to one side of the LepI pocket (Fig. 2b, Supplemental Fig. 6a, b) and maintains the catalytic domain in the active state through interaction networks. The adenosine rings and amino acids of SAM interact with Phe276 and Asn275 via π–π interaction and salt bonding, respectively. O4 of the ribose and the tail amide of SAM form a pair of hydrogen bonds with the main chain of Gly227. In addition, Arg291 forms a pair of hydrogen bonds with the O atom and amino group of the SAM tail via the side chain and main chain, respectively. Specifically, a hydrogen bond forms between the S^+^-CH_3_ moiety and the main chain of Leu292. In addition, Ile293 also forms a hydrogen bond with C5 of SAM. Collectively, these interactions result in the coordination of SAM in an extended confirmation (Fig. 2c).

Additional electron density was observed in the pocket of the LepI density map, located opposite the SAM-binding site. As MTA could be detected via HPLC analysis of the purified proteins compared with the reference standard of SAM and MTA (Supplemental Fig. 3a, b), we modeled MTA in the electron-dense cluster and found it likely to be coordinated in the hydrophobic site (Supplemental Fig. 6c), with some residues displaying significant conformational changes upon MTA binding (Fig. 2e, Supplemental Fig. 6d). Consistent with the structural observations, limited proteolysis assays indicated that purified LepI treated with MTA exhibited increased trypsin resistance, whereas SAH seemingly decreased trypsin resistance (Fig. 2f). We postulated that the MTA-binding site is the primary channel for substrates or products. The co-factor SAM is too remote from the substrate-binding site; thus, we speculated the electrostatic influence of the SAM sulfonium moiety for catalysis is limited.

**Fig. 3:**
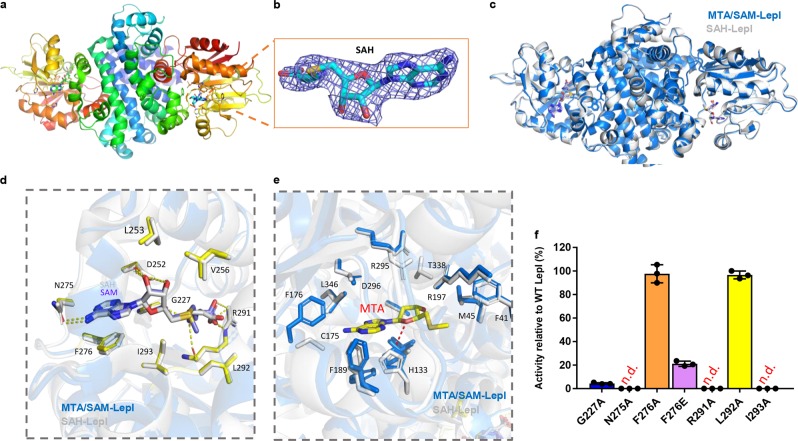
Activity regulation by SAH/SAM. **a** Schematic of the overall structure of LepI in complex with SAH. **b** The density map of SAH. The 2Fo–Fc omit map, contoured at 1.5σ. The SAH molecule is shown in stick. **c** Structural comparison of MTA/SAM-LepI (blue) and SAH-LepI (gray). **d** Close-up view of the SAM site between structures of MTA/SAM-LepI (blue) and SAH-LepI (gray). Interactions between SAM and residues (G227, N275, and F276) of LepI are indicated by yellow dashed lines. **e** Close-up view of the substrate site from MTA/SAM-LepI (blue) and SAH-LepI (gray) structures based on the overall structural alignment. MTA is located at the hydrophobic channel. **f** Enzymatic assays of the key residues around the SAM-binding site with variants, n.d. represents no detection of activity, (data represent the mean ± s.d.)

To better understand the mechanism underlying LepI inhibition by SAH, we determined the structure of SAH-bound LepI at a 2.7 Å resolution (Fig. 3a, b and Extended Data Table 1). The overall architectures of SAM/MTA-bound LepI and SAH-bound LepI seemed similar (Fig. 3c). SAM or SAH binding with the enzyme occurs at the inner surface of the pocket and is primarily mediated via ionic bonds, hydrogen bonds and π–π interactions (Fig. 3d). No hydrogen bonds form between Leu292 or Ile293 and SAH because of the absence of a methyl group, which decreases the binding affinity between the region (amino-acid residues 291–296) and SAH. However, based on structural observation, one additional salt bond appears to form between Arg291 and SAH. The close similarity between these two complexes revealed that the reactivity of LepI might result from the hydrogen bonding and positively charged nature of SAM vs SAH, but not related to structural reason.

### LepI activation and inhibition

Structural analysis in the present study revealed that SAM or SAH occupied a site on the inner surface of the pocket of LepI that was distinct from the MTA substrate-binding area (Figs. 2b, 3e, Supplemental Fig. 6a). Thermal unfolding assays revealed that SAM, SAH, and MTA increased the melting temperature (*T*m) of LepI by ~ 6.2 °С, 4.3 °С, and 4.4 °С, respectively (Supplemental Fig. 7a, b). Consistent with the structural observations and thermal assays, limited proteolysis assays indicated increased trypsin resistance in purified LepI in the presence of SAM or MTA, whereas SAH had negligible effects (Fig. 2f). Furthermore, MTA increased trypsin resistance to a greater extent than did SAM, suggesting that SAM can most likely stabilize LepI for activation, whereas MTA mimicked the substrate in the active site.

To validate the SAM-binding sites, we individually mutated the residues involved in SAM-binding via enzymatic assays. Concurrent with the results from structural analyses, Gly227Ala (G227A) substitution greatly decreased LepI activity, whereas Phe276 and Leu292 substitutions showed only a small loss in activity compared with wild-type LepI, and no activity was detected from Asn275Ala, Arg291Ala, and Ile293Ala, as no protein was obtained (Fig. 3f). These substitutions confirmed the importance of residues at the SAM-binding site, as revealed via the complex structure.

As shown in Fig. 1, MTA-and SAH-decelerated LepI activity; however, the underlying mechanisms remain unclear. MTA probably mimics the substrate binding at the active site, thereby blocking the entry of a native substrate as a competitive inhibitor (Fig. 1b), thereby decreasing LepI activity. Although SAH-mediated LepI inhibition probably proceeds via the failure of a stable interaction between SAH and LepI. Applying SAH and MTA simultaneously almost obliterated LepI activity, indicating that SAH and MTA have synergistic effects with different underlying inhibitory mechanisms (Fig. 1c).

Overall, the present results suggest that SAM may stabilize one active state among many available LepI conformations through substrate mimicry, thereby activating the pericyclic reaction; however, SAH cannot completely activate the pericyclic reaction of LepI because it lacks of sulfonium moiety for catalysis, whereas MTA occupies the substrate site.

### Mechanism underlying LepI catalysis via the pericyclic reaction

**Fig. 4:**
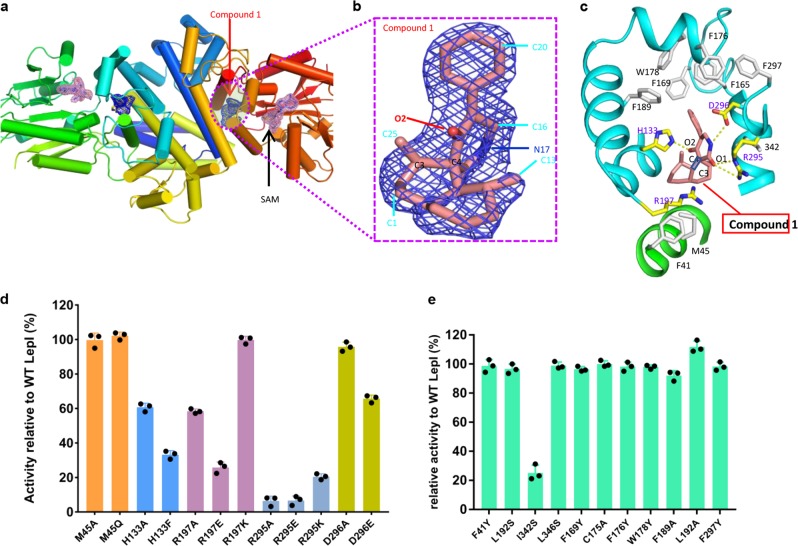
LepI catalyzes one step of the pericyclic reactions. **a** Overall structure of LepI in complex with SAM and the precursor of Leporin C compound **1**. Density maps are presented for SAM and precursor **1**, which are colored in pink and blue, respectively. **b** The *2Fo–Fc* omit map, contoured at 1σ. The compound **1** molecule is shown in stick representation. **c** Close-up view of the substrate-binding site. The compound was coordinated at the substrate site, where four polar residues for substrate binding and catalysis surrounded by hydrophobic bulky residues with a semiopen configuration (Supplemental Fig. 8). **d** Enzymatic assays of the mutants around the substrate-binding site. H133A, R197A, and R295A greatly impaired the enzymatic activity. **e** Mutation of Ile342 to serine greatly impaired the enzymatic activity, but most hydrophobic residues had little effect on the enzymatic activity

To further explore the catalytic mechanism of LepI, we determined the structure of the LepI-substrate complex at 2.2 Å (Fig. 4a, b, Extended Data Table. 1). The compound **1** was present at the substrate site, which is a hydrophobic pocket with a semiopen configuration (Fig. 4c, Supplemental Fig. 8). Three positive residues (His133, Arg197, and Arg295) and one negative residue (Asp296) are present along with some bulky hydrophobic residues (Phe41, Phe165, Phe169, Phe176, Trp178, and Phe189) around the compound, coordinated in a curve confirmation via salt bonds, hydrogen bonds, and π–π interactions (Fig. 4c). The polar residue Arg197 is proximal to the C3–C4 double bond, and this positively charged residue has been proposed to decrease C4 electron density of the olefin in favor of potential attack by the 2′-carbonyl oxygen. Arg295 forms hydrogen bonds with an oxygen atom (1′O) of the carbonyl group of compound **1** and may be involved in providing a positively charged electrostatic environment for the reaction; moreover, Asp296 also interacts with the compound **1** via hydrogen bonding (Fig. 4c). Notably, His133 not only forms an instant hydrogen bond with 2′-carbonyl oxygen but also probably forms a π-stacking interaction with the imidazole ring (Fig. 4c). Simultaneously, the active site is formed with the assistance of the bulky hydrophobic residues including Phe41, Phe165, Phe169, Phe176, Trp178, and Phe189, which form π–π and hydrophobic interactions to maintain a hydrophobic environment. Therefore, to induce the retro-Claisen rearrangement reaction, His133, Arg295, and Asp296 participate in coordinating the substrate in the chair conformation with the assistance of other residues; thereafter, together with Arg197, they provide electrostatic charges to catalyze the rearrangement (Fig. 4c).

**Fig. 5:**
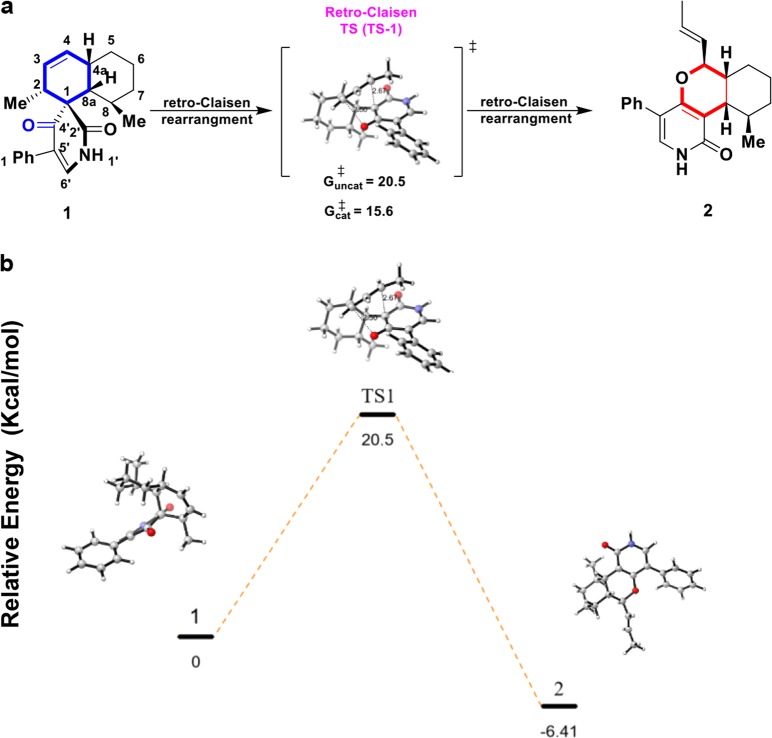
DFT-computed free energies for the retro-Claisen rearrangement reactions. **a** Summary of LepI-catalyzed reaction cascade leading from **1** to **2**. **b** Free-energy diagram are shown for the non-enzymatic formation of **2** from **1**. calculated with B3LYP-D3/6–31 G(d), gas phase. Numbers on levels show Gibbs free energies in kcal mol^−1^

To verify important residues around the substrate, we performed an enzymatic assay using a panel of LepI variants harboring amino-acid substitutions within the substrate-binding site (Fig. 4d, e). Consistent with structural observations, replacing the substrate-interacting residues with alanine significantly reduced catalytic activity. Specifically, mutating His133, Arg197, and Arg295 to alanine, and Asp296 to glutamic acid impaired enzymatic activity (Fig. 4d). Similar results were obtained upon mutation of Ile342 to serine (I342S; Fig. 4e), where this mutation potentially converted a hydrophobic residue to a polar residue to thereby alter the catalytic environment. Notably, mutating the bulky residue Phe41 (F41Y), which packs against the phenolic moiety of compound **1**, retained appreciable catalytic activity. We individually replaced other bulky hydrophobic residues (Phe165, Phe169, Phe176, Trp178, Phe189, and Phe297) adjacent to the substrate with alanine or tyrosine. We could not obtain the protein of these variants (Phe41Ala, Phe169Ala, Phe176Ala, and Phe297Ala). These results suggested that these residues formed a hydrophobic site and prevented other molecules, including water, from entering randomly, thereby maintaining a hydrophobic habitat. To further investigate the role of positively charged residues (Arg197 and Arg295), we performed the DFT calculation by using ^+^NH_2_ = CH_2_ as a simpler mimic of arginine side chain. The barrier (TS-1) of the retro-Claisen rearrangement of **1** to the final HDA product **2** was substantially lowered by 4.9 kcal mol^−1^, which proved the catalytic role of the positively charged residues around the substrate (Fig. 5a, b, Extended Data Table. 2). Thus, we propose that LepI may facilitate retro-Claisen rearrangement through a combination of the following processes: (i) elimination of water molecules surrounding the substrate; (ii) stabilization of the reactive geometry, perhaps by decreasing the energy of retro-Claisen rearrangement via conformation immobilization; and (iii) enhancement of the reactivity by cationic residues.

## Discussion

The biochemical and structural analyses of the present study revealed that the functional evolution of a classic methyltransferase LepI in fungi and the catalytic role of LepI in retro-Claisen rearrangment reaction. The structure of SAM-bound LepI suggests the dimeric or oligomeric state is essential for enzymatic activity, as indicated via an enzymatic assay involving N-terminal deletion (Fig. 2d, e). SAM activates LepI, whereas MTA and SAH decrease LepI activity; however, SAH is a more-effective inhibitor than MTA, and MTA and SAH have synergistic effects on LepI inhibition when simultaneously present (Fig. 1b–d). These results imply that these inhibitions proceed through distinct mechanisms. Analyses of the LepI crystal structures in complex with different compounds revealed that SAM stabilizes the LepI structure and collaboratively participates the catalytic reaction as an activator, whereas SAH could partially inhibit the LepI activity as an inhibitor, probably owing to the leakage of the sulfonium cation, whereas MTA, occupied the substrate site, as a competitive inhibitor (Figs. 1b–d and 2b). These findings were supported by the biochemical analyses (Fig. 1b–d). The structural conformational changes of these structures (Fig. 3d, e) indicate that several residues with different confirmations are present in the structures (Supplemental Fig. 6d). Additional analysis of trypsin resistance showed that LepI stability may be further enhanced via MTA mimicry (Fig. 2f).

Positively charged residues interact with compound **1** and accelerate retro-Claisen rearrangement of IMDA adduct **1** to the final HAD product **2** (Supplemental Fig. 1), and the reaction is potentially accelerated 100–1000-fold by LepI, and SAH can decelerate LepI activity. However, our analyses suggest that the pericyclic reaction depends not only on the SAM but also on the presence of polar residues (His133, Arg197, Arg295, and Asp296) surrounding the substrate site, which allow substrate **1** to form preferentially in a lower-energy TS-1 configuration (Fig. 5a, b). Notably, analysis of enzyme kinetics revealed that SAM and SAH potentially alter the *K*_m_ of compound **1**, and several critical residues also largely influence *K*_m_ values (Supplemental Fig. 9), consistent with structural observations.

Together, the present structural and biochemical analyses elucidate the possible mechanism underlying LepI activation and extend our understanding of this enzyme, revealing a distinct regulatory mechanism. Similar SAM-dependent enzymes, most probably methyltransferases, have been studied extensively for decades, suggesting that the complexity of enzymatic mechanisms regulating activation and inhibition may have coevolved with fungi.

The present study reveals the novel catalytic mechanism underlying the pericyclic reaction of this enzyme. However, owing to limitations associated with intermediate structures, further studies are required to elucidate the detailed mechanisms underlying each catalytic step. Overall, our findings deepen our understanding of LepI enzymatic activity and provide a fundamental basis for the rational design of natural-product derivatives for use in drug design and development.

## Methods

### Chemicals

Compounds **1** and **3–5** were chemically synthesized according to the literature with minor modifications.^32^ NMR and HRMS spectroscopic data of Compound **2** was consistent with the report in the literature (Supplemental Fig. 10).^22^ Compound **1** was analyzed by MS spectroscopy and biochemical assay (Supplemental Fig. 11). HPLC-grade acetonitrile was purchased from Merck; water was purified and deionized by a water purification system from Biopak Milli-Q. All of the other chemicals used in this study were obtained from standard sources for laboratory use.

### Truncation constructs and mutations

The N-terminal deleted truncations, including LepI-Δ15 and LepI-Δ37, were designed based on the structure for disrupting the dimerization. The other mutations were performed for studying enzymatic activity. The primers for the mutations and truncations were all generated on the QuickChange Primer Design website, and the constructed plasmids were verified by Sanger sequencing.

### Gene cloning, protein expression, and purification

A codon-optimized gene encoding LepI, originated from *A. flavus*, was integrated into the expression vector pET-15b (Novagen, Madison, WI, USA) between the NdeI and XhoI sites with an N-terminal histidine tag. DNA sequencing and transformation into *Escherichia coli* BL21 (DE3) cells for expression verified the constructed plasmid pET-15b-LepI. A 100 ml overnight culture was used to inoculate 6 L LB liquid medium supplemented with the essential antibiotic Ampicillin. When the cell density reached an OD 600 nm of 0.6, the culture temperature was decreased from 37 °C to 15 °C, and the culture was supplemented with 0.3 mmol/L isopropyl-d-1-thiogalactopyranoside to induce the expression of LepI for 18 h. Cell pellets harvested by 4000 rpm centrifugation for 15 min were resuspended in 300 mL lysis buffer (25 mmol/L Tris-HCl pH 7.5, 150 mmol/L NaCl, 1 mmol/L phenylmethylsulfonyl fluoride) and disrupted using a low-temperature high-pressure cell disruptor. The lysate was clarified by centrifugation at 15,000 g for 45 min at 4 °C, and the supernatant was loaded onto a column containing 5 mL Ni resin. After an extensive wash with lysis buffer, the target protein LepI was eluted with elution buffer (25 mmol/L Tris-HCl pH 7.5, 150 mmol/L NaCl, 250 mmol/L imidazole). The elution was concentrated to 2 mL and was loaded directly onto a HiLoad Superdex 200 10/300 GL size-exclusion chromatography column (GE Healthcare) in sample buffer (10 mmol/L Tris-HCl pH 7.5, 100 mmol/L NaCl, 5 mmol/L dithiothreitol; DTT). The major peak fractions were analyzed with 12% sodium dodecyl sulfate polyacrylamide gel electrophoresis (SDS-PAGE). Selenomethionine LepI was expressed in M9 medium supplemented with SeMet (Generon) to a final concentration of 100 μg/mL and purified similarly to the native proteins.

### Crystallization, data collection, and structure determination

Purified LepI was concentrated to ~ 10 mg/mL in sample buffer (10 mmol/L Tris-HCl pH 7.5, 100 mmol/L NaCl, 5 mmol/L DTT). Crystallization conditions were initially determined with the sparse matrix screen (Hampton Research). Screening trials were established in 96-well sitting-drop plates by combining 1 μL of protein solution with an equal volume of well buffer. Crystals of LepI were grown in buffer containing 0.15 m bis–Tris (pH 7.0) and 40% PEG 400. The crystals grew to their maximum size within 7 days. The crystals were mounted in a nylon loop and directly flash-frozen in liquid nitrogen. The diffraction data were collected at the Shanghai Synchrotron Radiation Facility at beamline BL19U1 and BL17U1 using a CCD detector. The diffraction images of LepI were indexed, integrated and merged in XDS and scaled with SCALA as implemented in the xia2 package.^33–35^ Single-wavelength anomalous dispersion data were collected on a selenomethionine-substituted LepI crystal at the selenium absorption peak (0.97918 Å). The diffraction data were processed and scaled using HKL3000.^36^ The final model was manually constructed in COOT^37^ and refined in Phenix2.^38^ The SAH-LepI and compound **1**-bound LepI structures were solved with molecular replacement (Molrep, Phenix2) with the corresponding SAM/MTA-LepI structure, built in COOT, and refined by Phenix2. Data collection and processing statistics are summarized in online Table S1.

### Activity assays of LepI and its mutants

Assays for LepI activity with mixture containing compound **1** in phosphate buffer (20 mm Na_2_HPO_4_, 50 mm NaCl, pH 8.0) were performed at the 50 μL scale with 0.3 or 3 µm LepI at 30 °C for 10 min. Then, the reaction was quenched with a 3-equal volume of cold acetonitrile. Protein was precipitated and removed by centrifugation, and the supernatant was analyzed with an high-performance liquid chromatoggraphy (HPLC) system (Dionex UltiMate 3000, Thermo Scientific, USA) equipped with a LPG-3400SDN pump system, a WPS-3000SL auto sampler with a 20 μL injection loop, a TCC-3000RS column oven, and a VWD-3100 variable wavelength detector. A C18 column (Phenomenex Gemini-C18 column 150 mm × 4.6 mm, 5 μm) was used with isocratic conditions (40% of H_2_O in CH_3_CN). UV detection was performed at 260 nm. Final results were calculated as percentage of control values. The error bars in the figures represent the standard deviation (S.D.) of three independent replicates. Data fitting was performed using GraphPad Prism 6.

### Evaluation of SAH/MTA effects on retro-Claisen rearrangement

Various concentrations of SAH (0–1 mm) or MTA (0–1 mm) were added to 50 μL solutions containing LepI in phosphate buffer (20 mm Na_2_HPO_4_, 50 mm NaCl, pH 8.0) in the presence or absence of cofactors and incubated at RT for 10 min. Then, the mixture containing compound **1** was added to the reaction mixture to initiate the enzymatic reaction. After 10 min of incubation at 30 °C, reactions were quenched by the adding 3 × volume of acetonitrile. Protein was precipitated and removed by centrifugation, and the supernatant was analyzed by HPLC using a reversed-phase column ((Phenomenex Gemini-C18 column 150 mm × 4.6 mm, 5 μm) with isocratic conditions (40% of H_2_O in CH_3_CN). Results were compared to the activity of WT-LepI. Final results were calculated as percentage of control values. The error bars in the figures represent the standard deviation (S.D.) of three independent replicates. Data fitting was performed using GraphPad Prism 6.

### Analytic size-exclusion chromatography

Proteins used in the experiments, including LepI, LepI-T3A/L10A/Q13A/L14A, LepI-Δ15, and LepI-Δ37-GST, were purified as described above. Pure LepIs were all adjusted to a final concentration of 1 mg/mL before injection into an analytical gel­filtration column (Superdex 200 Increase 10/30 GL; GE Healthcare) equilibrated with buffer containing 20 mm Na_2_HPO_4_ and 50 mm NaCl (pH 8.0). The migration positions of the LepI variants were compared with the position of native-LepI. The standard shift was monitored by injecting the following known proteins: thyroglobulin (bovine) (670 kD), γ-globulin (bovine) (158 kD), ovalbumin (chicken) (44 kD), myoglobin (horse) (17 kD), and vitamin B12 (1.35 kD).

### Trypsin digestion assay

MTA, SAH, and SAM in 10 mm stock solution was added slowly to LepI to achieve a final concentration of 1 mm, and the mixture was incubated on ice for 10 min. Trypsin stock solutions were prepared in Tris-Mg solution (10 mg/mL) and twofold serially diluted to eight different concentrations in buffer containing 20 mm Na_2_HPO_4_ and 50 mm NaCl (pH 8.0). Then, the trypsin was added to a final protease-to-protein ratio of 1:5 (v/v) and incubated on ice for 30 min. Loading buffer was added to the mixture to a final protease-to-ratio of 1:1 (v/v) at 100 °C for 10 min, and the mixture was then analyzed via 15% SDS-PAGE.

### Sequence alignment

Multiple sequence alignments were generated on the ClustalW online service and edited using the ESPript 3.0 program.^39^

## Supplementary information

Extend Data Table 2

Supplemental Figures

Extend Data Table 1

## Data Availability

The coordinates of the structures have been deposited in the Protein Data Bank (PDB) under accession codes 6J1O for MTA/SAM-LepI, 6J24 for SAM/ligand **1**-bound LepI, and 6J46 for SAH-LepI. Other data that support the findings of this study are available from the corresponding author upon request.
