# Supplementary material for: Deciphering the regulatory and catalytic mechanisms of an unusual SAM-dependent enzyme
Source: Signal Transduct Target Ther. 2019 May 24;4:17. doi: 10.1038/s41392-019-0052-y (PMC6533283; doi:10.1038/s41392-019-0052-y)
Supplement: Supplementary file 1 — Extend Data Table 2 [file 41392_2019_52_MOESM1_ESM.pdf]

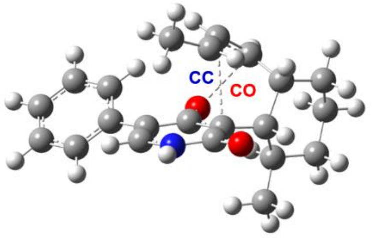

	TS-1		
	ΔG‡(Kcal/mol)	CC(Å)	CO(Å)
uncatalyzed	20.5	2.67	2.50
$NH_2^+{=}CH_2$	15.6	2.38	2.82
$NH_3^+CH_3$	15.1	2.45	2.83
$NH{=}CH_2$	20.0	2.61	2.59
NH_2-CH_3	23.4	2.65	2.52

Extended data Table 2 | Calculated free energies and bond distances for retro-Claisen rearrangement reaction from 1 to 2. Data are shown for the transition state for the retro-Claisen rearrangement (TS-1), uncatalysed and with a $NH_2^+{=}CH_2$ as a simpler mimic positive-charged arginine catalyzed reaction, calculated with B3LYP-D3/6-311+ G(d,p)//6-31G(d), CPCM water. Positions of the bonds are shown in the structures above.

Computational details

All DFT calculations were conducted with the Gaussian 16 software package[1]. Geometry optimizations and frequency calculation of all the minima and transition states were performed using B3LYP[2-4] functional with 6-31G(d) basis set, including Grimme's dispersion corrections[5]. The solvation energies were evaluated by a self-consistent reaction field (SCRF) using B3LYP functional with 6-311+G(d,p) basis set and the CPCM model[6]. The 3D diagrams of molecules were generated using GaussView.

Cartesian coordinates for calculated species

1

Charge = 0 Multiplicity = 1

C	-1.31764	-0.82656	0.15471
C	-2.23771	-1.7226	-0.74514
C	-2.72726	-1.01332	-1.98157
C	-2.76497	0.30877	-2.13669
C	-2.26409	1.29246	-1.11657

C	-1.96384	0.62264	0.2528
C	-1.12213	2.15831	-1.70128
C	-0.58532	3.15964	-0.67406
C	-1.25306	1.59433	1.24395
C	-0.1262	2.42422	0.59006
C	-2.29541	2.50014	1.92627
C	-1.42423	-1.39617	1.58065
C	0.1178	-0.76872	-0.40547
C	1.00998	-1.25325	1.80601
O	-2.48919	-1.66603	2.1124
O	0.29774	-0.66697	-1.61252
N	-0.24361	-1.57209	2.2827
C	1.25146	-0.8209	0.54363
C	2.62062	-0.4279	0.13608
C	3.1712	-0.81211	-1.09907
C	3.40403	0.35231	1.00485
C	4.47118	-0.43972	-1.43777
C	4.70624	0.7173	0.66576
C	5.24564	0.32098	-0.55887
C	-1.59335	-3.07902	-1.08015
H	-3.09055	1.99166	-0.90636
H	-2.94196	0.38958	0.69023
H	-1.50759	2.68542	-2.58424
H	-0.31212	1.51256	-2.04915
H	0.25224	3.72543	-1.10055
H	-1.36294	3.89503	-0.42404
H	-0.79256	1.00164	2.04459
H	0.71391	1.77113	0.32604
H	0.26208	3.13619	1.3306
H	-3.01621	1.90104	2.49428
H	-2.85923	3.09779	1.20194
H	-3.09676	-1.66044	-2.77647
H	-3.14611	0.73411	-3.06453
H	1.81541	-1.39327	2.51976

H	2.57441	-1.39593	-1.78772
H	2.97525	0.69686	1.9428
H	4.88219	-0.74896	-2.39521
H	5.29197	1.32358	1.35177
H	6.25751	0.60979	-0.8302
H	-0.35394	-1.93187	3.22284
H	-0.737	-2.95915	-1.74872
H	-1.25958	-3.59423	-0.17086
H	-2.32421	-3.72754	-1.57602
H	-3.11456	-1.93253	-0.11855
H	-1.81029	3.19584	2.62111

TS1

Charge = 0 Multiplicity = 1

C	-0.85421	0.83424	-0.35559
C	-0.45457	1.15418	2.26494
C	-0.56284	-0.21551	2.46452
C	-1.56044	-0.96065	1.85146
C	-2.66049	-0.30941	1.11355
C	-2.2175	0.22733	-0.35466
C	-3.96037	-1.13713	1.055
C	-3.95363	-2.21579	-0.02844
C	-2.38248	-0.84823	-1.47322
C	-3.73663	-1.56649	-1.39632
C	-2.1938	-0.19652	-2.85048
C	-0.72973	2.25971	-0.59037
C	0.28896	-0.03604	-0.27492
C	1.69963	1.88862	-0.71221
O	-1.65252	3.07975	-0.61545
O	0.11599	-1.25152	0.01515
N	0.59083	2.69527	-0.77891
C	1.62873	0.55429	-0.44737
C	2.86158	-0.26053	-0.35815
C	2.99633	-1.27402	0.60811

C	3.9485	-0.01517	-1.21566
C	4.18475	-1.99284	0.72423
C	5.13888	-0.73087	-1.09405
C	5.26406	-1.72284	-0.1201
C	0.75786	1.94242	2.63498
H	-2.89871	0.62103	1.63973
H	-2.93303	1.03205	-0.55815
H	-4.78904	-0.44602	0.84945
H	-4.15764	-1.5694	2.04413
H	-3.15299	-2.94474	0.16412
H	-4.89961	-2.77098	-0.00386
H	-1.60338	-1.601	-1.33332
H	-3.78272	-2.32169	-2.19181
H	-4.55194	-0.85279	-1.59458
H	-1.19094	0.22929	-2.95805
H	-2.91925	0.61238	-3.00779
H	0.27492	-0.74195	2.91603
H	-1.54807	-2.04232	1.94256
H	2.64845	2.39705	-0.84766
H	2.15626	-1.50157	1.25202
H	3.84992	0.72833	-2.00271
H	4.26786	-2.76978	1.48019
H	5.96308	-0.52295	-1.7717
H	6.18811	-2.2874	-0.02784
H	0.68821	3.68687	-0.95621
H	1.63476	1.30677	2.78684
H	0.98887	2.67682	1.85554
H	0.57042	2.51331	3.55693
H	-1.3283	1.73879	1.99811
H	-2.33442	-0.93529	-3.6486

1-NH_2Me

Charge = 0 Multiplicity = 1

C	1.06136	-0.44727	-0.80254

C	1.71357	-1.27387	-1.96228
C	1.70333	-2.75959	-1.70866
C	1.56506	-3.32703	-0.51171
C	1.33943	-2.5628	0.76301
C	1.56988	-1.03717	0.58411
C	-0.00553	-2.96091	1.41633
C	-0.25379	-2.1875	2.71495
C	1.14458	-0.22151	1.84381
C	-0.20126	-0.67882	2.44839
C	2.27308	-0.25164	2.89168
C	1.67741	0.96149	-0.86114
C	-0.47773	-0.47391	-0.89824
C	-0.52509	1.93462	-0.57672
O	2.89062	1.13566	-0.95598
O	-1.05179	-1.51457	-1.19988
N	0.83274	2.04112	-0.74987
C	-1.21896	0.76625	-0.5991
C	-2.67737	0.75335	-0.33882
C	-3.56597	-0.02747	-1.09895
C	-3.2035	1.54299	0.69932
C	-4.93466	0.00155	-0.83553
C	-4.57361	1.57555	0.9555
C	-5.44631	0.80384	0.18689
C	1.12233	-0.92354	-3.33889
H	2.12099	-2.8798	1.47398
H	2.65434	-0.91122	0.4825
H	0.01207	-4.04156	1.61149
H	-0.8228	-2.78035	0.71376
H	-1.23073	-2.45736	3.13493
H	0.49717	-2.46659	3.46772
H	1.0244	0.83029	1.55563
H	-1.0211	-0.41712	1.76941
H	-0.38073	-0.11705	3.37497
H	3.18857	0.19681	2.48781

H	2.51724	-1.27214	3.20588
H	1.84926	-3.38602	-2.58829
H	1.57876	-4.4129	-0.42243
H	-1.03259	2.88481	-0.43938
H	-3.17598	-0.65713	-1.88804
H	-2.5253	2.11632	1.32676
H	-5.60633	-0.6062	-1.43637
H	-4.9559	2.19165	1.76533
H	-6.51412	0.82028	0.38842
H	1.31194	2.96415	-0.69314
H	0.08712	-1.2632	-3.42526
H	1.15231	0.15856	-3.5174
H	1.70661	-1.40513	-4.13118
H	2.76282	-0.95025	-1.97475
H	1.98691	0.31039	3.78871
N	2.75714	4.0595	-0.35079
H	2.91605	5.03329	-0.60205
H	3.35461	3.48097	-0.94099
C	3.10722	3.81732	1.0576
H	2.421	4.37183	1.70624
H	2.98557	2.74911	1.26044
H	4.13714	4.09889	1.32277

TS1-NH_2Me

Charge = 0 Multiplicity = 1

C	-0.50939	0.53772	-0.20388
C	-0.10058	0.5903	2.41683
C	0.03939	-0.79175	2.44392
C	-0.83589	-1.61917	1.75604
C	-2.05197	-1.0815	1.11652
C	-1.7469	-0.29475	-0.27094
C	-3.19606	-2.10368	0.96071
C	-3.05095	-3.00971	-0.2618
C	-1.76563	-1.22728	-1.52163

C	-2.98765	-2.1552	-1.52859
C	-1.72423	-0.37887	-2.80065
C	-0.63365	1.97849	-0.26144
C	0.76982	-0.12488	-0.25521
C	1.8151	2.05561	-0.44962
O	-1.6827	2.63359	-0.18001
O	0.81693	-1.3775	-0.13016
N	0.5819	2.65743	-0.39846
C	1.9824	0.70692	-0.36273
C	3.33824	0.11303	-0.39955
C	3.67794	-0.97742	0.42142
C	4.33456	0.65096	-1.23293
C	4.97414	-1.48967	0.4227
C	5.6322	0.14105	-1.22611
C	5.9593	-0.93156	-0.39499
C	0.96482	1.52963	2.87517
H	-2.4291	-0.27886	1.75904
H	-2.59763	0.38504	-0.36411
H	-4.13486	-1.54166	0.86573
H	-3.28134	-2.69223	1.88282
H	-2.13626	-3.61499	-0.18179
H	-3.89282	-3.71177	-0.30387
H	-0.87354	-1.85684	-1.49021
H	-2.94564	-2.79216	-2.42159
H	-3.91092	-1.55911	-1.61286
H	-0.80482	0.21231	-2.86141
H	-2.57158	0.31835	-2.84161
H	0.9712	-1.21221	2.81485
H	-0.63354	-2.68465	1.70615
H	2.65648	2.73593	-0.52701
H	2.91348	-1.42712	1.04236
H	4.08206	1.46174	-1.91171
H	5.21588	-2.33123	1.06708
H	6.38342	0.57305	-1.88233

H	6.96807	-1.33578	-0.39308
H	0.50041	3.66485	-0.45285
H	1.94844	1.05336	2.91409
H	1.02136	2.39965	2.21214
H	0.71899	1.91526	3.87621
H	-1.07376	1.0364	2.24254
H	-1.77361	-1.01872	-3.68962
N	-4.62649	1.91967	-0.91117
H	-4.60428	1.07553	-1.48135
H	-3.6674	2.26608	-0.86632
C	-5.07727	1.59652	0.44147
H	-5.10414	2.51565	1.03709
H	-6.09842	1.1984	0.4069
H	-4.45414	0.86863	0.99534

1-NH_3^+Me

Charge = 1 Multiplicity = 1

C	0.85735	-0.65419	0.08901
C	1.71655	-1.39533	1.18004
C	2.15436	-0.49139	2.30436
C	2.13544	0.84301	2.27618
C	1.62194	1.66225	1.12632
C	1.42207	0.80758	-0.15407
C	0.415	2.53437	1.54973
C	-0.12733	3.3506	0.37193
C	0.70689	1.58946	-1.29738
C	-0.49066	2.42922	-0.79852
C	1.73022	2.45406	-2.05612
C	1.09545	-1.39695	-1.22728
C	-0.62319	-0.60162	0.53245
C	-1.3086	-1.56966	-1.58683
O	2.23431	-1.61405	-1.68778
O	-0.88398	-0.27534	1.6788
N	0.02032	-1.82783	-1.92616

C	-1.67915	-0.94046	-0.44903
C	-3.0982	-0.60321	-0.19624
C	-3.70056	-0.81536	1.05567
C	-3.86977	-0.05958	-1.2385
C	-5.04586	-0.50563	1.24608
C	-5.21499	0.24734	-1.04308
C	-5.80709	0.02318	0.20108
C	1.03934	-2.67504	1.70583
H	2.41713	2.37771	0.85741
H	2.43858	0.60442	-0.51262
H	0.74305	3.20028	2.35741
H	-0.37279	1.90113	1.96195
H	-1.01271	3.91439	0.68585
H	0.61657	4.09413	0.05419
H	0.31379	0.86593	-2.02415
H	-1.30559	1.76629	-0.4845
H	-0.88229	3.01228	-1.64097
H	2.50312	1.82495	-2.52063
H	2.23056	3.17428	-1.40034
H	2.51311	-1.00495	3.19546
H	2.46316	1.40113	3.15179
H	-2.03104	-1.92345	-2.31317
H	-3.11671	-1.22267	1.87118
H	-3.40343	0.14832	-2.19852
H	-5.50217	-0.68082	2.21603
H	-5.79542	0.67163	-1.85722
H	-6.85414	0.26569	0.35777
H	0.22347	-2.3186	-2.79037
H	0.1693	-2.43951	2.32185
H	0.71986	-3.32407	0.88132
H	1.74545	-3.24336	2.32004
H	2.62297	-1.73156	0.65726
H	1.24678	3.02104	-2.85826
N	4.65084	-0.60239	-1.20817

H	3.6614	-1.01263	-1.30291
C	5.15194	-0.64381	0.20885
H	4.4374	-0.1202	0.84794
H	5.21807	-1.68836	0.51437
H	6.13438	-0.17308	0.259
H	5.26492	-1.12649	-1.83982
H	4.60557	0.35985	-1.55803

TS1-NH_3^+Me

Charge = 1 Multiplicity = 1

C	-0.51126	0.42469	0.13302
C	-0.10774	-0.09573	2.4894
C	-0.03035	-1.48635	2.26939
C	-0.99084	-2.13419	1.53897
C	-2.1631	-1.39932	1.01576
C	-1.75294	-0.38016	-0.16485
C	-3.36087	-2.28563	0.62423
C	-3.21032	-2.94603	-0.74635
C	-1.73805	-1.05106	-1.5715
C	-3.01016	-1.87412	-1.81884
C	-1.55275	-0.00123	-2.67373
C	-0.65169	1.83648	0.35358
C	0.80305	-0.15333	-0.18732
C	1.76262	2.05956	0.1258
O	-1.74167	2.45413	0.58432
O	0.90291	-1.36763	-0.42487
N	0.50247	2.57573	0.3639
C	1.97619	0.74469	-0.13953
C	3.34388	0.23869	-0.39497
C	3.76907	-0.99356	0.13095
C	4.25903	1.00608	-1.13532
C	5.07898	-1.42844	-0.06065
C	5.5685	0.56843	-1.32575
C	5.98397	-0.64987	-0.78542

C	1.02401	0.67084	3.08835
H	-2.50656	-0.72748	1.81161
H	-2.596	0.32001	-0.16399
H	-4.26175	-1.65409	0.60919
H	-3.52743	-3.03165	1.4099
H	-2.34929	-3.62884	-0.74745
H	-4.09434	-3.55565	-0.96418
H	-0.88615	-1.73246	-1.60785
H	-2.95021	-2.33123	-2.81371
H	-3.89117	-1.20859	-1.83685
H	-0.62458	0.56636	-2.55636
H	-2.38848	0.71894	-2.6836
H	0.89156	-2.00242	2.52387
H	-0.86005	-3.18325	1.28743
H	2.57052	2.77873	0.19303
H	3.06732	-1.61169	0.67755
H	3.93519	1.93997	-1.58807
H	5.39363	-2.38118	0.35588
H	6.25958	1.17292	-1.90627
H	7.00255	-0.99515	-0.93716
H	0.38809	3.56411	0.55296
H	1.99835	0.25586	2.81909
H	0.99098	1.72732	2.80932
H	0.92388	0.63174	4.18431
H	-1.08323	0.37218	2.57532
H	-1.53463	-0.47415	-3.66093
N	-3.86728	2.55815	-0.8437
H	-3.75908	2.05722	-1.73035
H	-2.91812	2.46045	-0.27448
H	-4.00139	3.54916	-1.06277
C	-5.00819	2.01634	-0.0416
H	-5.0326	2.54123	0.91394
H	-5.94788	2.15804	-0.57741
H	-4.83124	0.95362	0.12938

1-NHCH$_2$

Charge = 0 Multiplicity = 1

C	0.90745	-0.79918	0.14053
C	1.71585	-1.67847	1.15668
C	1.98482	-0.97639	2.46301
C	1.94398	0.34281	2.64026
C	1.56815	1.32589	1.56769
C	1.51794	0.67451	0.15861
C	0.31137	2.1342	1.97253
C	-0.09416	3.13771	0.88826
C	0.95609	1.65211	-0.91818
C	-0.30047	2.41972	-0.4508
C	2.06301	2.62229	-1.3768
C	1.22933	-1.34616	-1.25816
C	-0.59601	-0.79619	0.48412
C	-1.14037	-1.20807	-1.85032
O	2.36488	-1.62679	-1.6244
O	-0.95618	-0.75605	1.65294
N	0.17927	-1.4981	-2.1383
C	-1.57612	-0.8256	-0.62618
C	-2.997	-0.46349	-0.41494
C	-3.71948	-0.8997	0.70925
C	-3.65333	0.34011	-1.36395
C	-5.06137	-0.55399	0.86151
C	-4.99712	0.67862	-1.21135
C	-5.70722	0.23094	-0.09636
C	1.08569	-3.06588	1.37004
H	2.38785	2.05992	1.50003
H	2.56057	0.47965	-0.11291
H	0.52624	2.65576	2.91477
H	-0.5169	1.45078	2.17485
H	-1.01746	3.65359	1.17972
H	0.67734	3.91322	0.78739

H	0.67079	1.06981	-1.80399
H	-1.14733	1.7315	-0.34838
H	-0.58424	3.13814	-1.23133
H	2.92419	2.08414	-1.78639
H	2.43024	3.24254	-0.55215
H	2.25367	-1.62556	3.29568
H	2.16035	0.7636	3.62163
H	-1.823	-1.33104	-2.68464
H	-3.22347	-1.50274	1.45857
H	-3.09415	0.72455	-2.21347
H	-5.60622	-0.90307	1.73468
H	-5.48308	1.3042	-1.95538
H	-6.75274	0.49887	0.02996
H	0.43447	-1.83968	-3.05701
H	0.13376	-2.99405	1.90185
H	0.91682	-3.57562	0.41336
H	1.75978	-3.69451	1.96254
H	2.6848	-1.8447	0.668
H	1.68034	3.29945	-2.14977
N	4.73269	0.25534	-1.81325
H	3.94022	-0.3391	-2.08278
C	5.28423	-0.2398	-0.78104
H	4.95399	-1.15976	-0.27913
H	6.14834	0.26622	-0.33959

TS1-NHCH$_2$

Charge = 0 Multiplicity = 1

C	-0.58208	-0.32644	0.5989
C	-0.47196	-1.35219	-1.80942
C	-0.35121	-0.0842	-2.37695
C	-1.18043	0.9507	-1.98572
C	-2.30044	0.72104	-1.05352
C	-1.81559	0.51511	0.48082
C	-3.4412	1.75156	-1.15921

C	-3.17064	3.05513	-0.40759
C	-1.69431	1.85723	1.26822
C	-2.92453	2.75364	1.07159
C	-1.4675	1.57052	2.75919
C	-0.67397	-1.59524	1.28691
C	0.69713	0.27057	0.27636
C	1.77741	-1.69986	1.20017
O	-1.71343	-2.16523	1.66629
O	0.73749	1.38568	-0.29918
N	0.55072	-2.22058	1.53081
C	1.92038	-0.50254	0.5701
C	3.26599	0.00539	0.21854
C	3.49631	0.67347	-0.99771
C	4.35941	-0.20272	1.07728
C	4.77893	1.09128	-1.3478
C	5.64293	0.21145	0.72343
C	5.8596	0.85877	-0.49399
C	0.57187	-2.40816	-1.96221
H	-2.73076	-0.25723	-1.28991
H	-2.63961	-0.0488	0.93165
H	-4.34558	1.28904	-0.7402
H	-3.65693	1.94255	-2.21818
H	-2.29007	3.5612	-0.82974
H	-4.0189	3.74004	-0.53184
H	-0.828	2.39776	0.88073
H	-2.77863	3.68484	1.63477
H	-3.81585	2.26629	1.49804
H	-0.52839	1.03313	2.92657
H	-2.28021	0.9587	3.17238
H	0.53862	0.1478	-2.95801
H	-0.99554	1.95216	-2.36467
H	2.62643	-2.32879	1.44589
H	2.65901	0.87602	-1.65353
H	4.1966	-0.67261	2.04405

H	4.93486	1.60313	-2.29417
H	6.47085	0.04113	1.40699
H	6.85769	1.18922	-0.76922
H	0.48324	-3.10453	2.01881
H	1.55147	-1.99489	-2.21872
H	0.6663	-2.99332	-1.04131
H	0.26788	-3.11351	-2.75074
H	-1.4376	-1.69141	-1.44923
H	-1.4261	2.50611	3.32986
N	-3.55212	-2.33823	-0.60028
H	-3.04066	-2.38679	0.29403
C	-4.78279	-2.61135	-0.45462
H	-5.25222	-2.88667	0.50035
H	-5.45034	-2.58133	-1.32185

1-$NH_2^+CH_2$

Charge = 1 Multiplicity = 1

C	0.90789	-0.66283	0.07893
C	1.7663	-1.3803	1.18781
C	2.27604	-0.44032	2.25042
C	2.2912	0.89346	2.16308
C	1.74259	1.67849	1.00602
C	1.47613	0.7869	-0.23673
C	0.56535	2.57646	1.46214
C	-0.03216	3.35909	0.28857
C	0.7051	1.54208	-1.36412
C	-0.46157	2.40367	-0.83086
C	1.69055	2.37939	-2.20012
C	1.12465	-1.4529	-1.21206
C	-0.56541	-0.5751	0.53615
C	-1.2834	-1.60846	-1.54181
O	2.2583	-1.70275	-1.66985
O	-0.80681	-0.19362	1.67006
N	0.03901	-1.88484	-1.89208

C	-1.63572	-0.94417	-0.41773
C	-3.05	-0.59523	-0.15463
C	-3.63275	-0.76624	1.11272
C	-3.8368	-0.0829	-1.20126
C	-4.97425	-0.44769	1.31462
C	-5.17814	0.23316	-0.99429
C	-5.7509	0.04962	0.26551
C	1.05567	-2.60218	1.80248
H	2.53213	2.37599	0.67985
H	2.46845	0.56768	-0.64881
H	0.93979	3.2649	2.22993
H	-0.20395	1.96208	1.93373
H	-0.89604	3.93914	0.63149
H	0.69872	4.08601	-0.09084
H	0.27311	0.79919	-2.0478
H	-1.26511	1.76015	-0.45439
H	-0.89084	2.96391	-1.67034
H	2.44232	1.73812	-2.6771
H	2.21979	3.12197	-1.59366
H	2.65268	-0.92359	3.15143
H	2.66572	1.47764	3.00264
H	-2.01772	-1.97895	-2.24756
H	-3.03684	-1.14881	1.93157
H	-3.3853	0.09416	-2.17443
H	-5.41543	-0.59094	2.29674
H	-5.77054	0.63318	-1.81206
H	-6.79485	0.2995	0.43097
H	0.22753	-2.40213	-2.74419
H	0.22057	-2.29957	2.43666
H	0.67805	-3.2741	1.02228
H	1.7613	-3.17118	2.41653
H	2.6419	-1.7901	0.66602
H	1.1635	2.92019	-2.99296
N	4.70739	-1.00242	-0.98498

H	5.39299	-1.2924	-1.68336
C	5.08416	-0.38593	0.06775
H	4.34049	-0.08585	0.81138
H	6.13785	-0.16668	0.22728
H	3.67583	-1.24611	-1.18887

TS1-$NH_2^+CH_2$

Charge = 1 Multiplicity = 1

C	-0.57789	0.37603	0.16742
C	-0.20444	-0.0957	2.46908
C	-0.06799	-1.49222	2.28873
C	-0.99223	-2.19125	1.5636
C	-2.18611	-1.50781	1.01511
C	-1.80168	-0.46039	-0.14548
C	-3.33498	-2.44591	0.59809
C	-3.14104	-3.07495	-0.78184
C	-1.75211	-1.10408	-1.56518
C	-2.98692	-1.97559	-1.83412
C	-1.60267	-0.02645	-2.64635
C	-0.74478	1.78965	0.34912
C	0.75389	-0.16885	-0.16497
C	1.65827	2.07272	0.10791
O	-1.84749	2.39829	0.57817
O	0.88598	-1.37937	-0.39343
N	0.38437	2.5583	0.34117
C	1.904	0.75996	-0.13575
C	3.28191	0.28435	-0.39316
C	3.74153	-0.92739	0.15099
C	4.17138	1.06184	-1.15396
C	5.06053	-1.33227	-0.04313
C	5.48984	0.6537	-1.34738
C	5.93979	-0.54403	-0.78892
C	0.89	0.72027	3.07672
H	-2.57621	-0.8613	1.81112

H	-2.66847	0.2106	-0.14221
H	-4.26513	-1.86064	0.58619
H	-3.4671	-3.21326	1.36984
H	-2.24709	-3.71432	-0.78797
H	-3.99272	-3.72307	-1.01684
H	-0.87524	-1.75234	-1.60867
H	-2.90101	-2.41124	-2.83677
H	-3.89601	-1.34995	-1.84388
H	-0.6876	0.56177	-2.52379
H	-2.45391	0.67317	-2.63533
H	0.87059	-1.96324	2.5685
H	-0.82211	-3.2404	1.33683
H	2.44569	2.8154	0.15776
H	3.06	-1.55298	0.71451
H	3.82044	1.97899	-1.62048
H	5.40248	-2.26909	0.38764
H	6.16103	1.2647	-1.94412
H	6.96554	-0.86631	-0.94285
H	0.24242	3.54825	0.50199
H	1.88303	0.35096	2.80939
H	0.81205	1.77634	2.80602
H	0.78755	0.66666	4.17154
H	-1.20094	0.32481	2.56439
H	-1.57622	-0.47953	-3.6427
N	-3.97399	2.38727	-0.72769
H	-4.12851	1.86365	-1.58978
H	-2.96568	2.28582	-0.16918
C	-4.89088	3.15808	-0.29616
H	-4.71264	3.71336	0.62268
H	-5.83923	3.27455	-0.81818

2

Charge = 0 Multiplicity = 1

C	-3.26128	1.32364	-1.0188

C	-4.05885	1.28947	0.29181
C	-4.2115	-0.14793	0.80404
C	-2.85779	-0.85976	0.98272
C	-2.0683	-0.83696	-0.36063
C	-1.90723	0.60058	-0.90976
C	-0.70083	-1.46621	-0.28209
C	0.42225	-0.68849	-0.10259
C	-0.88882	1.37001	-0.04908
C	-3.05455	-2.27603	1.53543
O	0.3799	0.65756	0.027
C	-0.55363	2.72664	-0.58893
C	-0.79783	3.86996	0.05433
C	-0.50664	5.2424	-0.47864
C	-0.57999	-2.88833	-0.52749
C	1.75576	-1.25143	-0.07007
C	1.84299	-2.60393	-0.25148
N	0.73958	-3.37266	-0.46878
O	-1.50216	-3.66762	-0.77686
C	2.9826	-0.44119	0.12505
C	4.11986	-0.68857	-0.66228
C	3.06222	0.55614	1.1125
C	5.3038	0.02038	-0.45891
C	4.24249	1.26955	1.30947
C	5.37021	1.00372	0.5287
H	-4.75685	-0.15555	1.75694
H	-3.55459	1.89947	1.05469
H	-5.04414	1.74679	0.13688
H	-3.84559	0.8177	-1.79953
H	-3.11135	2.35651	-1.35318
H	-2.27568	-0.29333	1.7263
H	-2.6539	-1.42239	-1.08184
H	-1.47061	0.52856	-1.91616
H	-4.82051	-0.72558	0.09191
H	-1.25555	1.46244	0.97933

H	-2.09925	-2.75094	1.77566
H	-3.65759	-2.24338	2.45137
H	-3.55773	-2.91749	0.80571
H	-0.09785	2.73455	-1.57948
H	-1.24702	3.82596	1.04844
H	-0.05167	5.20107	-1.47389
H	-1.42381	5.84278	-0.5467
H	0.17655	5.78591	0.18755
H	2.79277	-3.12557	-0.21679
H	0.82672	-4.3707	-0.61609
H	4.06541	-1.42883	-1.4564
H	2.19267	0.76908	1.72348
H	6.16923	-0.18737	-1.08289
H	4.28305	2.03459	2.08057
H	6.28887	1.56292	0.68454

Reference

1 Gaussian 16 Rev. A.03 (Wallingford, CT, 2016).

2 Becke, A. D. Density-functional thermochemistry. III. The role of exact exchange. **98**, 5648-5652, doi:10.1063/1.464913 (1993).

3 Lee, C., Yang, W. & Parr, R. G. Development of the Colle-Salvetti correlation-energy formula into a functional of the electron density. *Physical Review B* **37**, 785-789, doi:10.1103/PhysRevB.37.785 (1988).

4 Stephens, P. J., Devlin, F. J., Chabalowski, C. F. & Frisch, M. J. Ab Initio Calculation of Vibrational Absorption and Circular Dichroism Spectra Using Density Functional Force Fields. *The Journal of Physical Chemistry* **98**, 11623-11627, doi:10.1021/j100096a001 (1994).

5 Grimme, S., Antony, J., Ehrlich, S. & Krieg, H. A consistent and accurate ab initio parametrization of density functional dispersion correction (DFT-D) for the 94 elements H-Pu. *The Journal of Chemical Physics* **132**, 154104, doi:10.1063/1.3382344 (2010).

6 Barone, V. & Cossi, M. Quantum Calculation of Molecular Energies and Energy Gradients in Solution by a Conductor Solvent Model. *The Journal of Physical Chemistry A* **102**, 1995-2001, doi:10.1021/jp9716997 (1998).
