# Supplementary material for: Deciphering the regulatory and catalytic mechanisms of an unusual SAM-dependent enzyme
Source: Signal Transduct Target Ther. 2019 May 24;4:17. doi: 10.1038/s41392-019-0052-y (PMC6533283; doi:10.1038/s41392-019-0052-y)
Supplement: Supplementary file 2 — Supplemental Figures [file 41392_2019_52_MOESM2_ESM.pdf]

Supplemental Figures

Deciphering the regulatory and catalytic mechanisms of an unusual SAM-dependent enzyme

Qiu Sun[1]#, Yuehong Hu[1]#, Yijun Gu[2]#, Jiangkun Huang[3], Jun He[1], Lan Luo[3], Yi Yang[4], Shuo Yin[4], Chao Dou[1], Tianqi Wang[1], Xianghui Fu[1], Ling He[3], Shiqian Qi[1], Xiaofeng Zhu[1], Shengyong Yang[1], Xiawei Wei[1]*, Wei Cheng[1]*

1. Division of Respiratory and Critical Care Medicine, Center of Infectious Diseases, National Clinical Research Center for Geriatrics and State Key Laboratory of Biotherapy, West China Hospital of Sichuan University and Collaborative Innovation Center of Biotherapy, Chengdu, 610041, China.

2. Shanghai Synchrotron Radiation Facility, Zhangjiang Lab, Zhangheng Road 239, Pudong District, Shanghai, 201203, China.

3. Key Laboratory of Drug-Targeting and Drug Delivery System of the Education Ministry, Sichuan Engineering Laboratory for Plant-Sourced Drug and Sichuan Research Center for Drug Precision Industrial Technology, Department of Medicinal Chemistry, West China School of Pharmacy, Sichuan University, Chengdu, 610041, China

4. West China School of Public Health, Sichuan University, Chengdu, 610041, China

[#]These authors contributed equally to this work

*Corresponding author: Wei Cheng, chengwei669@scu.edu.cn; Xiawei Wei, xiaweiwei@scu.edu.cn

Figure S1

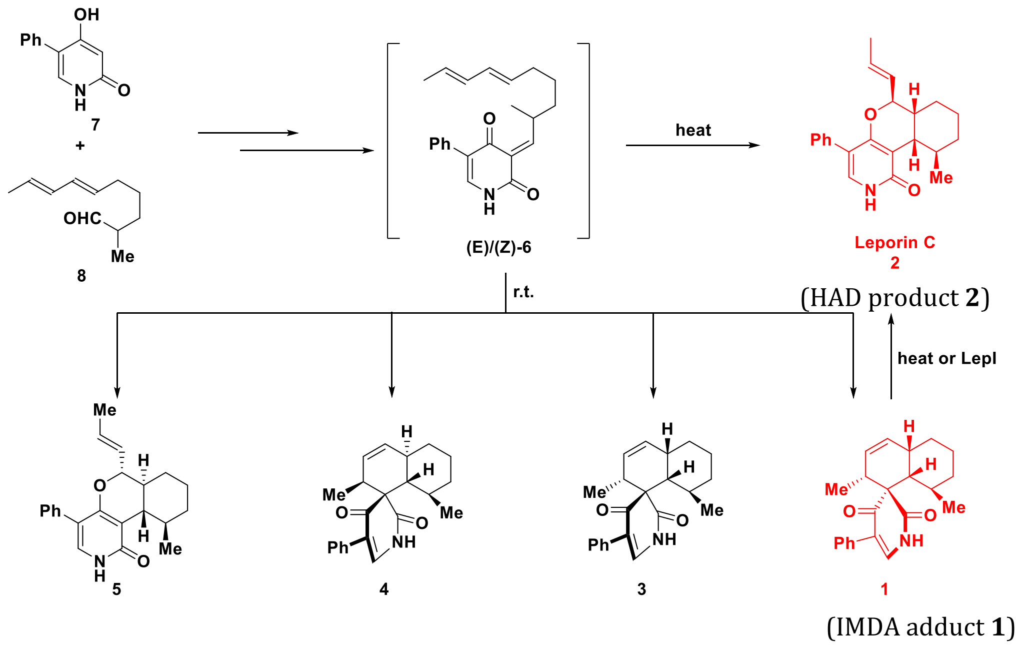

Figure S1. Total synthesis of Leporin C and its precursors. Synthetic route of Leporin C and its precursors. The structures show the relative stereochemistry[1].

Figure S2

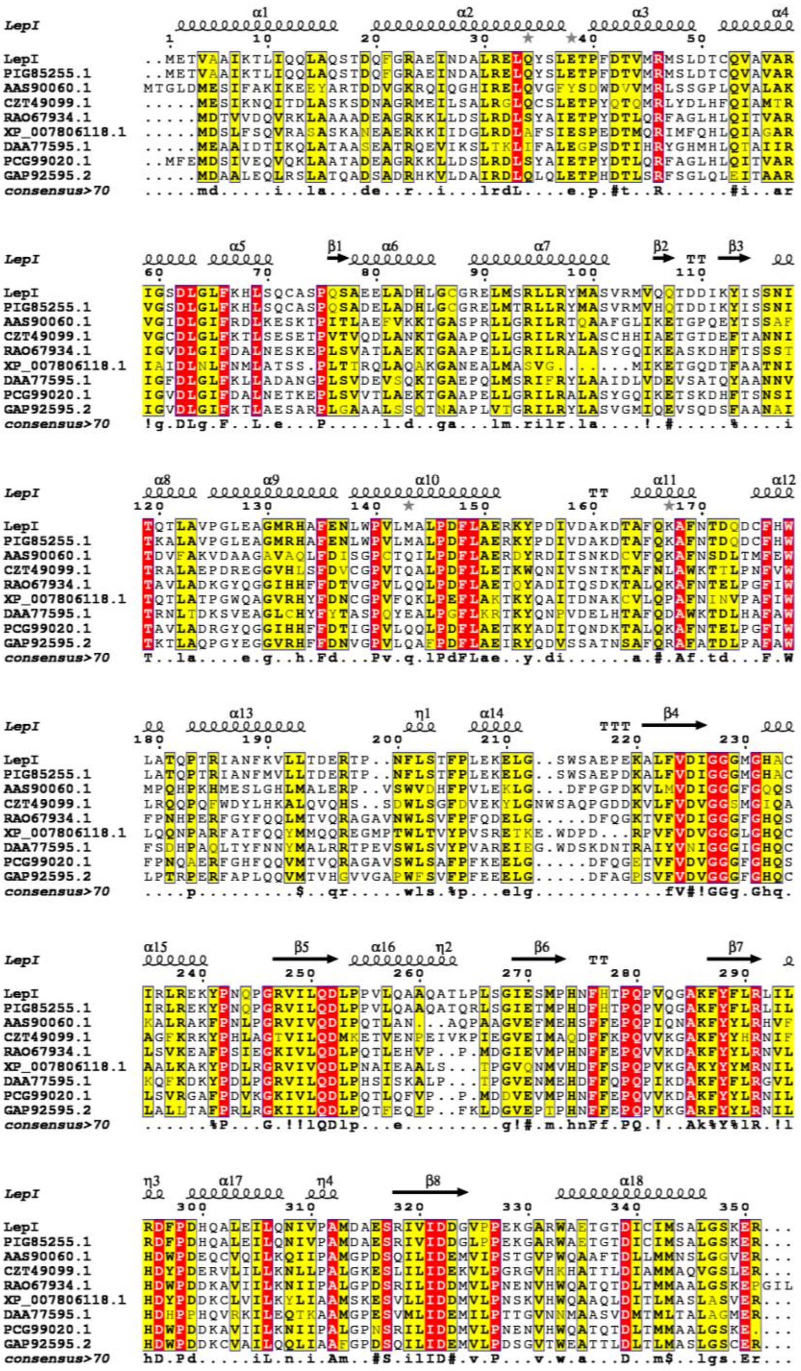

Figure 2S. Multiple sequence alignments of LepI in representative species. Conserved residues and invariant residues are indicated in yellow boxes and red boxes, respectively. Accession numbers are shown on the left. Secondary structure elements of LepI are shown on the top. Numbering is shown for the LepI sequence only. Multiple sequence alignments were generated on the ClustalW online service and were edited using the ESPript 3.0 program[2].

Figure S3

a

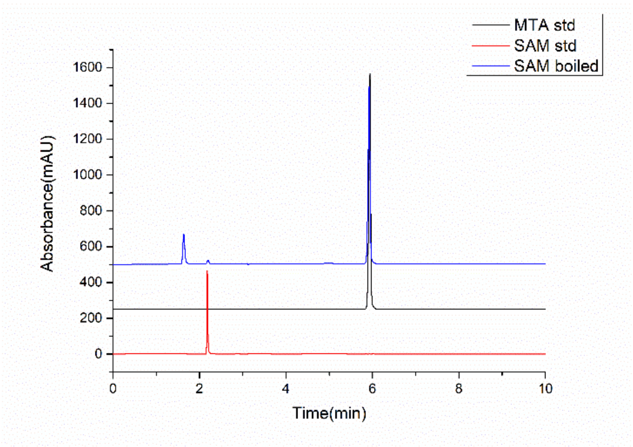

b

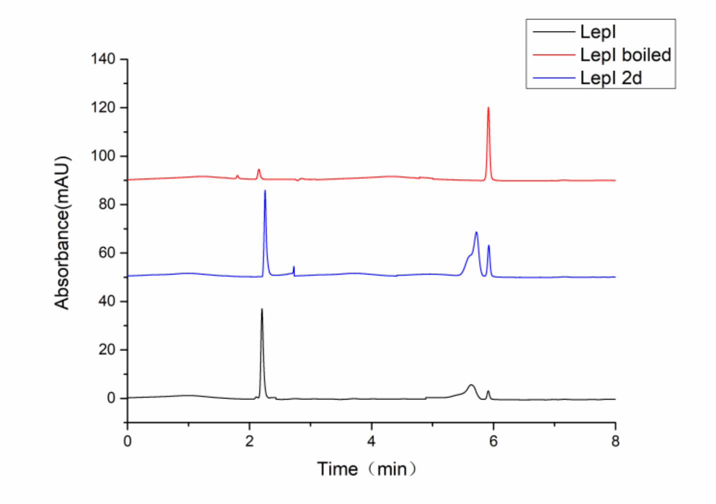

Figure S3. The confirmation of MTA in the present of LepI. (**a**) HPLC analysis of SAM and MTA. When SAM was boiled at 95°C for 10 min, it was decomposed to 5′ -deoxy-5′ -(methylthio) adenosine (MTA). (**b**) SAM and a tiny amount of MTA were detected in the supernatant of denatured (by acetonitrile) LepI. After LepI was maintained at 20°C for 2 days, an increased amount of MTA was observed. When LepI was denatured by heating the sample at 95 °C for 10 min, a single peak corresponding to MTA was detected from the supernatant of boiled LepI.

Figure S4

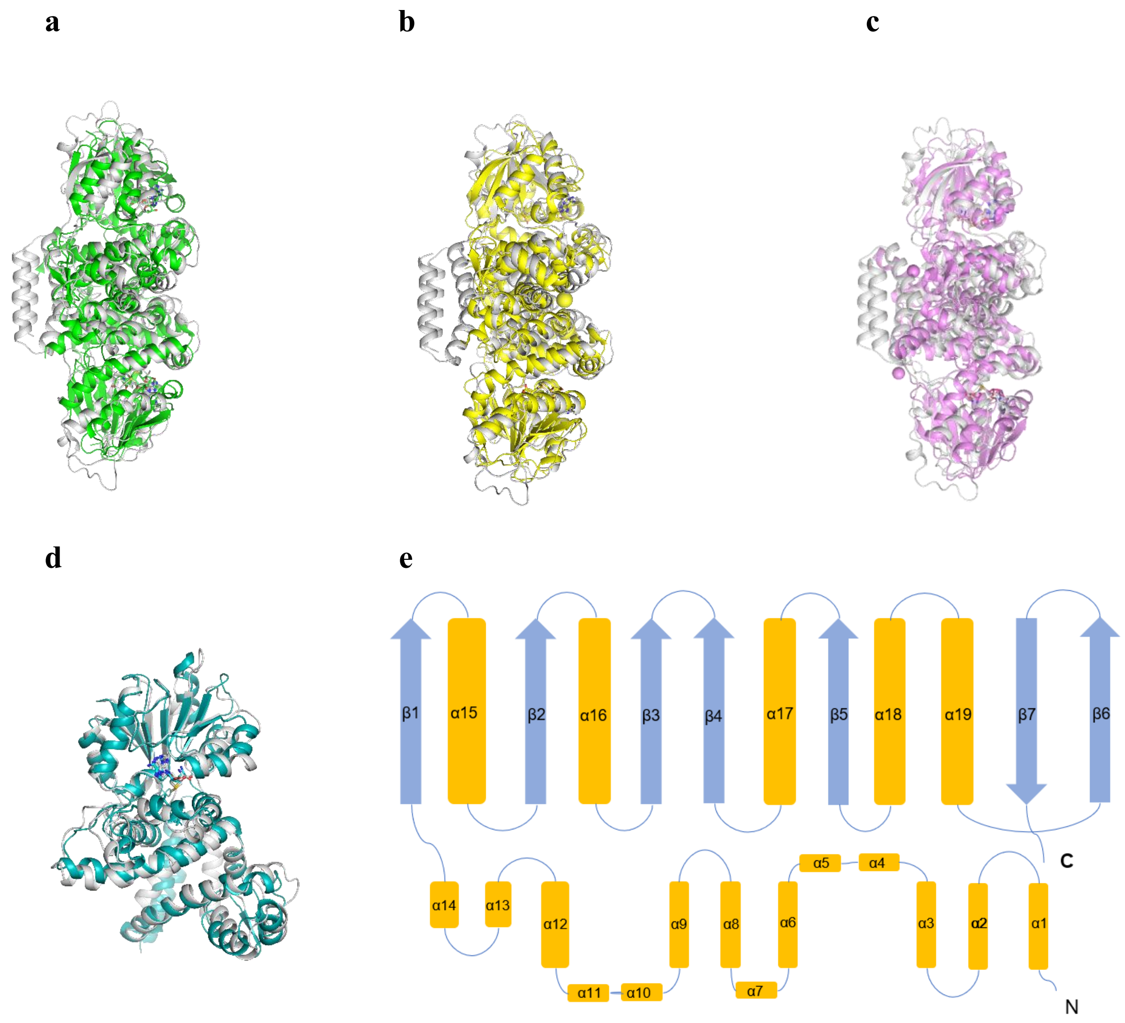

Figure S4. Structural comparison of LepI with representative homologues in SAM-dependent superfamily. (a) A ribbon presentation of monolignol o-methyltransferase (green) (PDB ID: 3TKY)[3] overlaid with LepI (light gray). A unique feature of LepI is a pair of N-terminal helices that comprise a 4-helix bundle in the protein dimer. **(b)** A ribbon presentation of LaPhzM (yellow) (PDB ID: 6C5B)[4] overlaid with LepI (light gray). A unique feature of LepI is a pair of N-terminal helices that comprise a 4-helix bundle in the protein dimer. **(c)** A ribbon presentation of mitomycin 7-O-methyltransferase MmcR (purple) (PDB ID: 3GWZ)[5] overlaid with LepI (light gray). A unique feature of LepI is a pair of N-terminal helices that comprise a 4-helix bundle in the protein dimer. **(d)** A ribbon presentation of OxaC (blue) (PDB ID: 5W7R)[6] overlaid with LepI (light gray). **(e)** Schematic showing the topology of SAM-dependent enzyme LepI. Helices are shown as yellow cylinders, and strands are shown as blue arrows. The N and C termini are labeled.

Figure S5

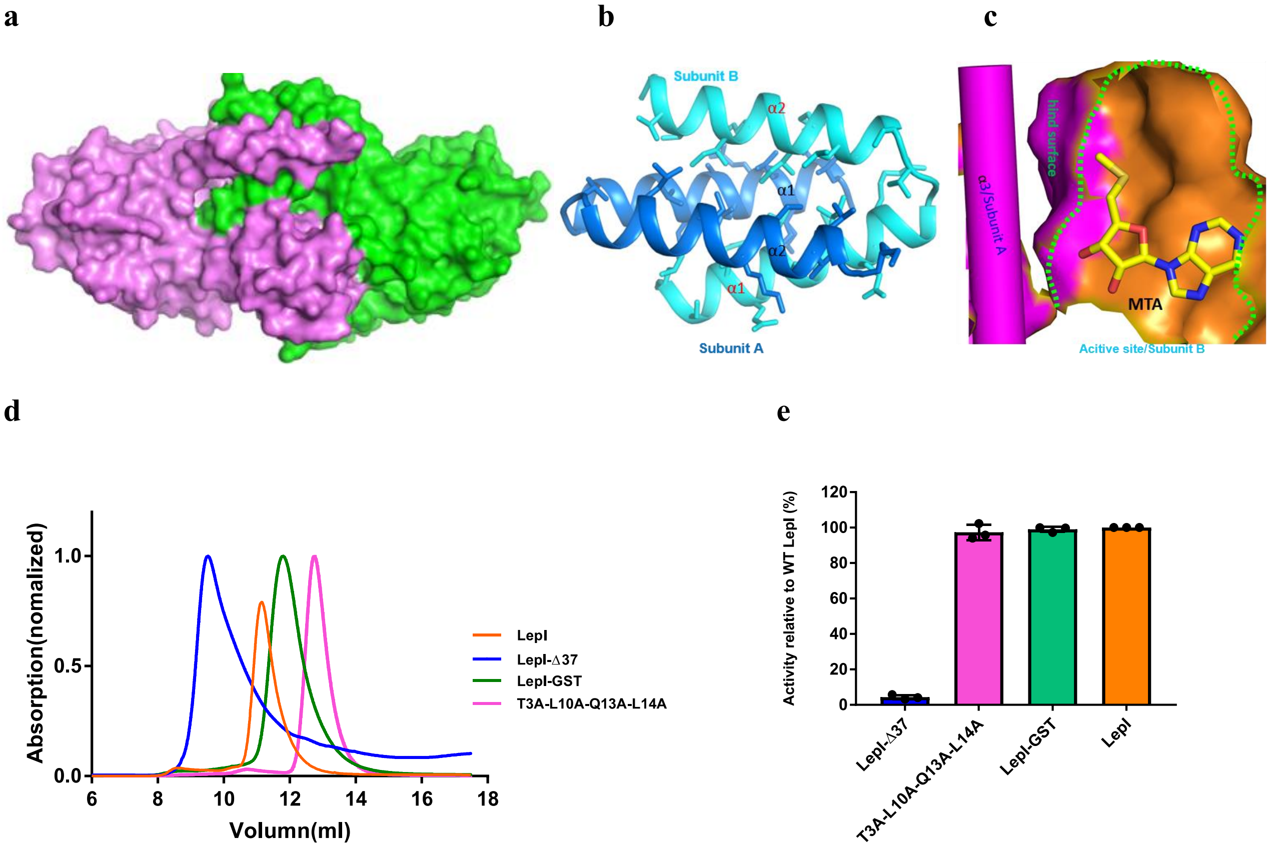

Figure S5 . N-terminal fragment is critical for the dimerization of LepI. (a) Surface representation of the LepI dimer. The two molecules are colored pink and green, respectively. **(b)** A close-up view of the N-terminal of LepI. The α1 and α2 segments of one subunit interact with those of another subunit to form interlocking fingers. **(c)** α3 is involved in the formation of the hind surface of the active site. **(d)** Analytic gel filtration of purified LepI-Δ15, LepI-Δ37, LepI-GST, LepI-T3A-L10A-Q13A-L14A and LepI. LepI-Δ37 shows aggregation. GST at the N-terminal and T3A-L10A-Q13A-L14A mutation disrupts the tetramer and forms a dimer. **(e)** Activity of LepI-Δ37, LepI-T3A-L10A-Q13A-L14A, LepI-GST compared to wild-type (WT) LepI through a retro-rearrangement assay with triplicate measurements. (Data represent means ± s.d.) The results indicate that LepI dimerization is essential for its activity.

Figure S6.

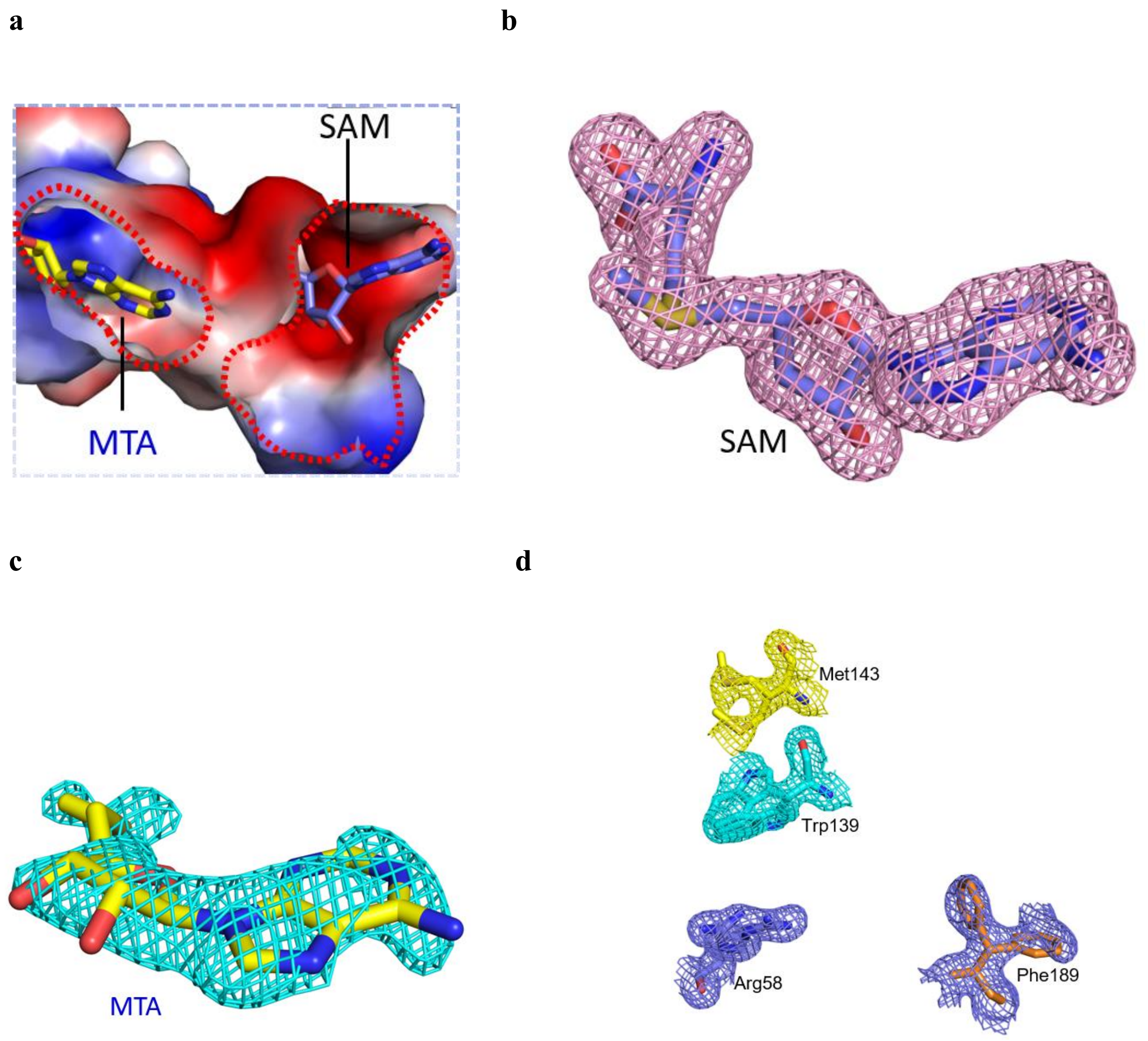

Figure S6. The binding surface and electron density maps of SAM and MTA. (a) Electrostatic potential surface of the binding pocket of SAM and MTA. SAM and MTA are indicated with different colors. **(b)** The 2Fo–Fc omit map, contoured at 1.5σ. The SAM molecule is shown in stick representation. **(c)** The 2Fo–Fc omit map, contoured at 1σ. The MTA molecule is shown in stick representation. **(d)** The representative residues display significant conformational changes upon MTA binding.

Figure S7.

a b

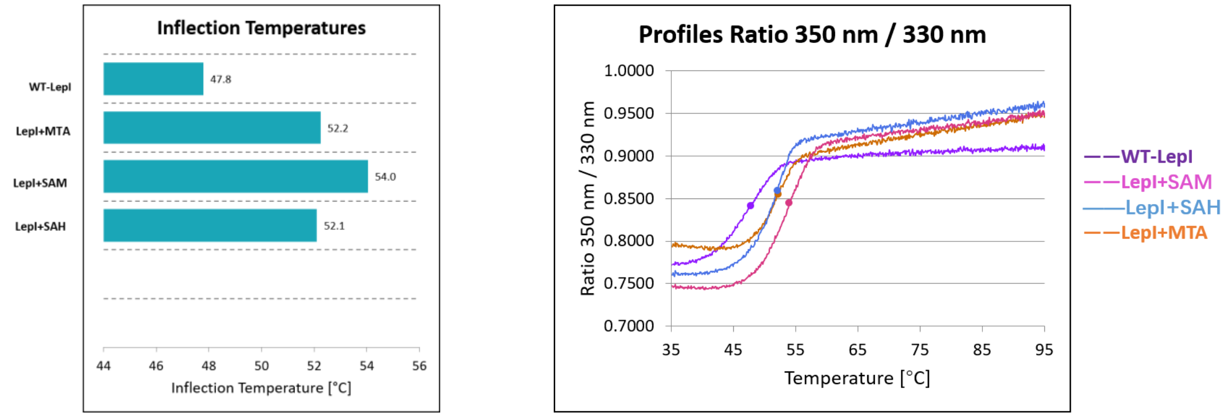

Figure S7. Stability of LepI in the absence or presence of MTA, SAM and SAH. (a) The thermal stability of LepI was measured by Nanotemper Prometheus NT.48. Thermal unfolding was performed in nanoDSF grade high-sensitivity glass capillaries (NanoTemper Technologies) at a heating rate of 1°C per minute. The inflection temperatures of LepI alone, LepI with 1 mM MTA, LepI with 200 µM SAM, and LepI with 200 µM SAH were 47.8°C, 52.2°C, 54.0°C, and 52.1°C, respectively. **(b)** The shift of intrinsic tryptophan fluorescence of proteins upon temperature-induced unfolding was monitored by detecting the emission fluorescence at 330 and 350nm. Infection temperature was calculated from the first derivative of the ratio of tryptophan emission intensities at 330 and 350 nm.

Figure S8.

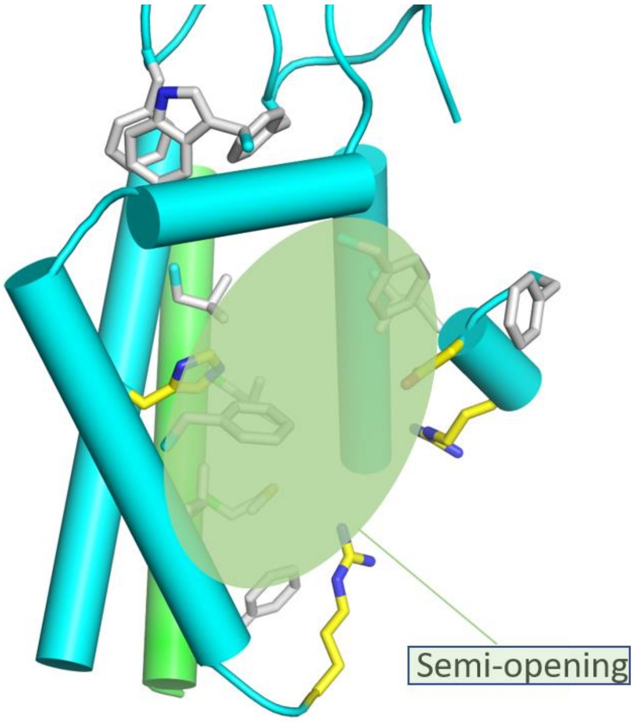

Figure S8. Substrate binding site is a hydrophobic pocket with a semi-open configuration. Close-up view of the substrate binding site. The residues involved in catalysis are shown in yellow stick representation, whereas the other hydrophobic residues are in grey stick representation.

Figure S9.

a

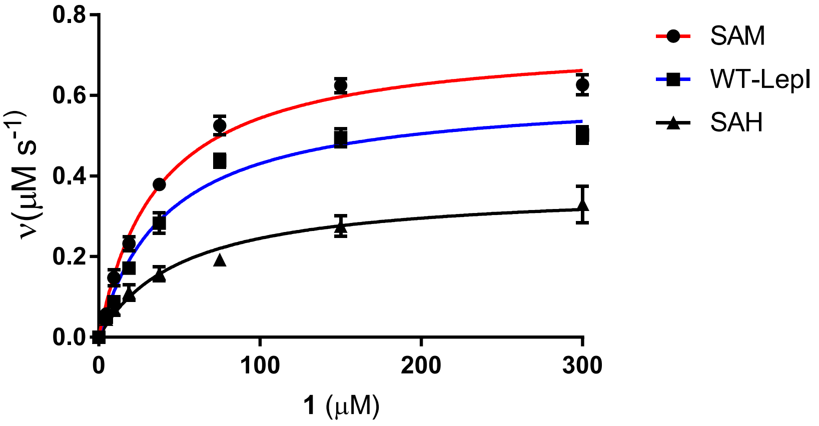

b

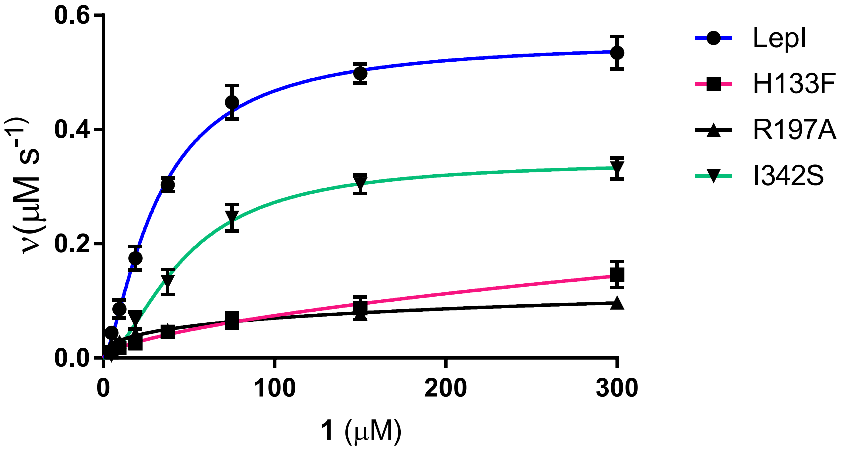

Figure S9. Kinetic analysis of LepI-catalyzed retro-Claisen rearrangement. (a) Rate versus estimated compound 1 concentration plot in the absence and presence of SAM or SAH (25μM). The Km value for WT-LepI, LepI(SAH) and LepI(SAM) is 41.58±4.32 μM, 51.34±7.74 μM, and 36.61±2.87 μM, respectively. Results are representative of three independent experiments. **(b)** Residues involved in LepI catalyzing retro-Claisen rearrangement were mutated to several analogs. R295A lost almost all the activity, so it could not be fitted with the Michaelis-Menten model (data not shown).

Figure S10.

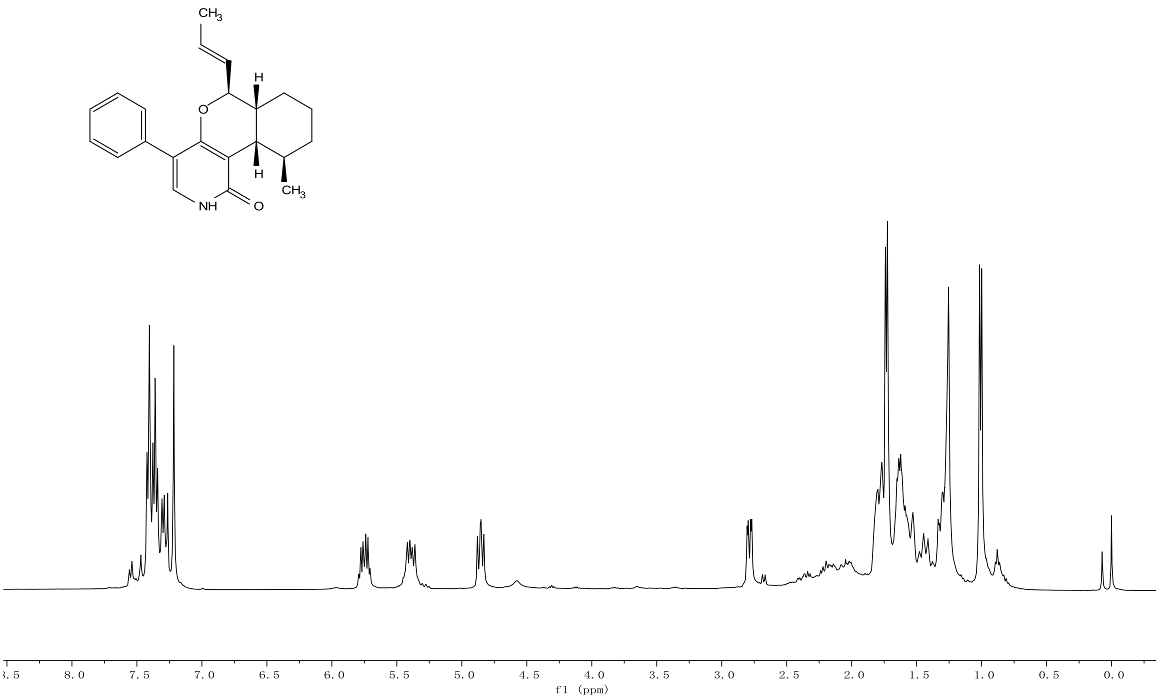

Figure S10. ^{1}H NMR spectra of compound **2**. Spectroscopic data of Leporin C (Compound **2**). ^{1}H NMR (400 MHz, Chloroform-d) δ 12.41 (s, 1H), 7.41 (d, *J = 7.6 Hz*, 2H), 7.36 (t, *J = 7.4 Hz*, 2H), 7.30 (d, *J = 7.0 Hz*, 1H), 7.22 (s, 1H), 5.75 (dq, *J = 13.3, 6.5 Hz*, 1H), 5.39 (dd, *J = 15.2, 8.2 Hz*, 1H), 4.85 (dd, *J = 11.4, 8.2 Hz*, 1H), 2.79 (dd, *J = 10.8, 3.8 Hz*, 1H), 1.91 – 1.30 (m, 11H), 1.01 (d, *J = 6.5 Hz*, 3H). HRMS (ESI, MH^+) calculated for $C_{22}H_{26}NO_2$ was 336.1958; the observed value was 336.1957. The spectral data are identical to those previously reported[7].

Figure S11.

a

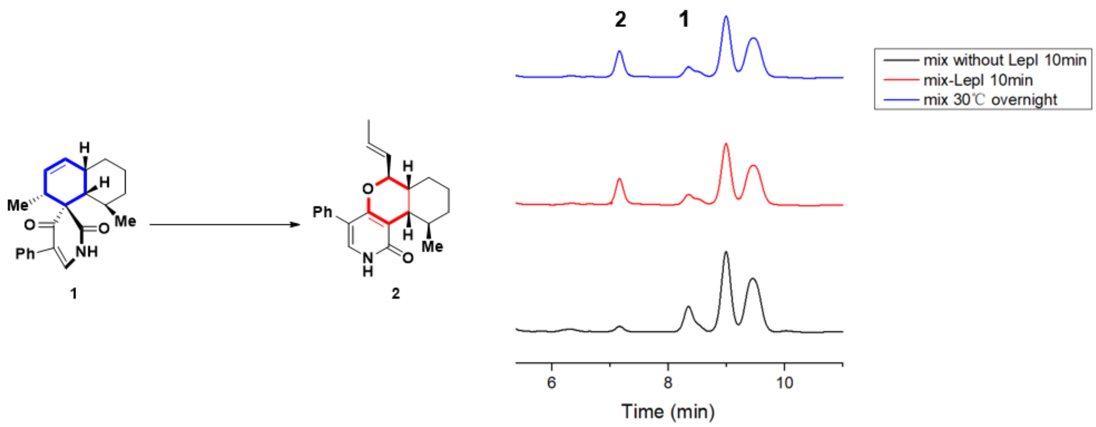

b

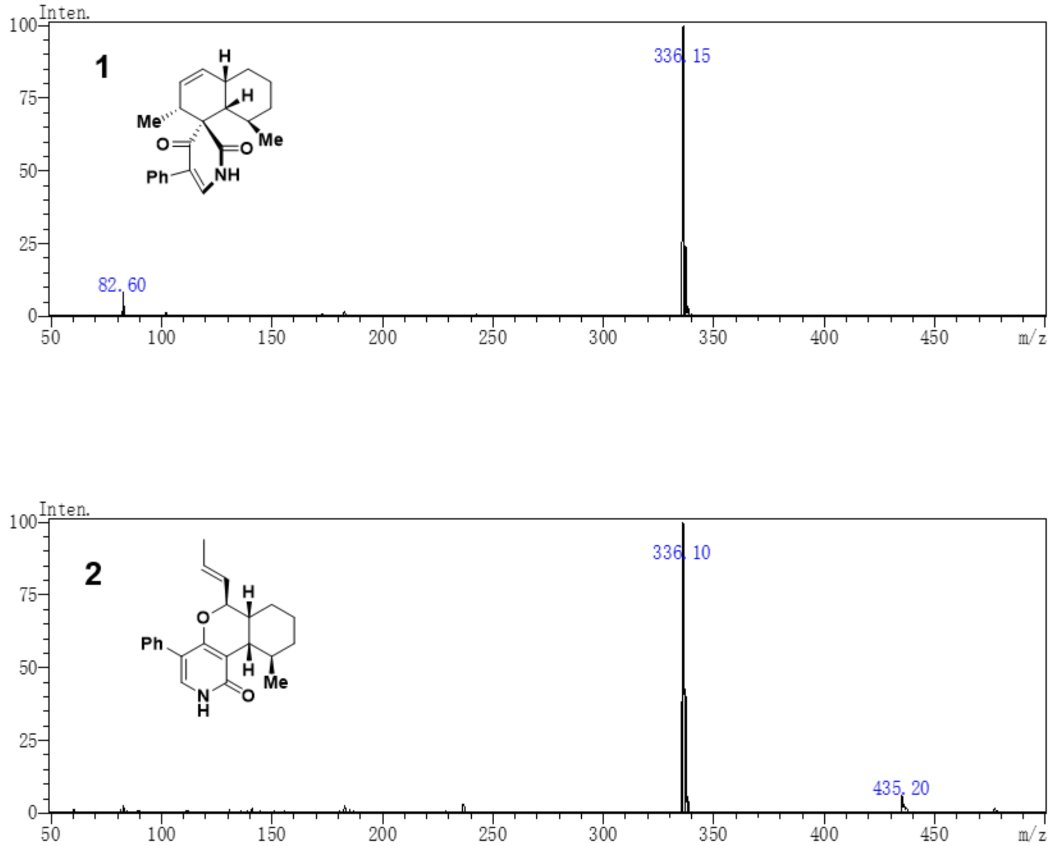

Figure S11. Confirmation of compound 1 in the mixture by HPLC assay and mass spectroscopy. (a) A mixture containing compound 1 (dissolved in 5% DMSO with H_2O) was reacted with LepI at 30°C for 10 min or without enzyme at 30°C overnight. Compound **1** was completely converted to **2** via [3,3]-sigma

tropic retro-Claisen rearrangement. This reaction is irreversible under these conditions. The structures show the relative stereochemistry. **(b)** Mass spectra of compound **1** and compound **2**. Instrument: Shimadzu LC-MS 8050 (Detector: triple four-stage bar); Chromatographic column: Sepax GP-C18 (50 x 2.1 mm, 1.8 um); Mobile phase: acetonitrile: 0.1% formic acid = 60:40; Column temperature: 40°C; Flow velocity: 0.2 mL/min; Scanning range of Q3 (+) scan mode: 50-500 m/z.

REFERENCE

1 Snider, B. B. & Lu, Q. Total synthesis of (+/-)-leporin A. *J Org Chem* **61**, 2839-2844, doi:DOI 10.1021/jo952053i (1996).

2 Larkin, M. A. *et al.* Clustal W and Clustal X version 2.0. *Bioinformatics* **23**, 2947-2948, doi:10.1093/bioinformatics/btm404 (2007).

3 Zhang, K. *et al.* An engineered monolignol 4-o-methyltransferase depresses lignin biosynthesis and confers novel metabolic capability in Arabidopsis. *Plant Cell* **24**, 3135-3152, doi:10.1105/tpc.112.101287 (2012).

4 Jiang, J. *et al.* Functional and Structural Analysis of Phenazine O-Methyltransferase LaPhzM from Lysobacter antibioticus OH13 and One-Pot Enzymatic Synthesis of the Antibiotic Myxin. *ACS Chem Biol* **13**, 1003-1012, doi:10.1021/acschembio.8b00062 (2018).

5 Singh, S. *et al.* Structural characterization of the mitomycin 7-O-methyltransferase. *Proteins* **79**, 2181-2188, doi:10.1002/prot.23040 (2011).

6 Newmister, S. A. *et al.* Unveiling sequential late-stage methyltransferase reactions in the

meleagrin/oxaline biosynthetic pathway. *Org Biomol Chem* **16**, 6450-6459, doi:10.1039/c8ob01565a (2018).

7 Ohashi, M. *et al.* SAM-dependent enzyme-catalysed pericyclic reactions in natural product biosynthesis. *Nature* **549**, 502-506, doi:10.1038/nature23882 (2017).
