# Supplementary material for: Deciphering the regulatory and catalytic mechanisms of an unusual SAM-dependent enzyme
Source: Signal Transduct Target Ther. 2019 May 24;4:17. doi: 10.1038/s41392-019-0052-y (PMC6533283; doi:10.1038/s41392-019-0052-y)
Supplement: Supplementary file 3 — Extend Data Table 1 [file 41392_2019_52_MOESM3_ESM.pdf]

Extended Data Table 1 │ Data collect and refinement statistics

	LepI-SAM/MTA<br>PDB ID:6J1O	LepI-SAH<br>PDB ID:6J46	LepI-ligand<br>PDB ID:6J24
**Data collect**			
Space group	C121	C121	C121
**Cell dimensions**			
a, b, c (Å)	160.24, 62.08, 133.62	159.82, 62.33, 112.73	160.44, 62.15, 112.30
α, β, γ (°)	90.00, 113.22, 90.00	90.00, 112.45, 90.00	90.00, 112.88, 90.00
**Resolution (Å)**	50.0– 1.70	104.19 - 2.62	50.0 – 2.24
**R_{-meas} (%)（inner/outer shell）**	4.5(2.9/30.9)	0.106(0.054/0.966)	6.8(3.6/75.0)
CC（1/2）	99.9%（99.9%/96.6%）	99.8% (99.7%/96.6%)	99.9% (99.9%/89.0%)
***I/σ* (*I*)**	22.7 (53.48/3.97)	12.4（26.1/2.6)	16.57 (43.88/2.53)
**Completeness (%)**	97.9(98.9/87.5)	92.2 (99.8/53.5)	99.3 (47.20-2.24)
**Redundancy**	5.83 (5.71/5.143)	6.6(6.3/5.9)	6.57(6.10/6.39)
**Refinement**			
**No. reflections**	140338	28684	49185
**R_{work}/R_{free} (%)**	18.09/19.95	20.87/24.56	19.77/22.65
**No. atoms**	6973	6202	6370
Protein	6273	6192	6236
Ligand/ion			
**Average B-factors**	24.6	63.3	49.6
**R.m.s deviations**			
Bond length (Å)	0.005	0.003	0.005
Bond angles (°)	0.853	0.583	0.810

Value in parentheses is for the lowest and highest-resolution shell.
